# High diversity of *Diaporthe* species associated with dieback diseases in China, with twelve new species described

**DOI:** 10.3897/mycokeys.39.26914

**Published:** 2018-09-17

**Authors:** Qin Yang, Xin-Lei Fan, Vladimiro Guarnaccia, Cheng-Ming Tian

**Affiliations:** 1 The Key Laboratory for Silviculture and Conservation of the Ministry of Education, Beijing Forestry University, Beijing 100083, P.R. China, V. Guarnaccia Beijing Forestry University Beijing China; 2 Westerdijk Fungal Biodiversity Institute, Uppsalalaan 8, 3584 CT, Utrecht, The Netherlands Westerdijk Fungal Biodiversity Institute Utrecht Netherlands; 3 Department of Plant Pathology, University of Stellenbosch, Matieland 7602, South Africa University of Stellenbosch Matieland South Africa

**Keywords:** Dieback, DNA phylogeny, Systematics, Taxonomy

## Abstract

*Diaporthe* species have often been reported as important plant pathogens, saprobes and endophytes on a wide range of plant hosts. Although several *Diaporthe* species have been recorded in China, little is known about species able to infect forest trees. Therefore, extensive surveys were recently conducted in Beijing, Heilongjiang, Jiangsu, Jiangxi, Shaanxi and Zhejiang Provinces. The current results emphasised on 15 species from 42 representative isolates involving 16 host genera using comparisons of DNA sequence data for the nuclear ribosomal internal transcribed spacer (ITS), *calmodulin* (*cal*), histone H3 (*his3*), partial translation elongation factor-1α (*tef1*) and β-tubulin (*tub2*) gene regions, as well as their morphological features. Three known species, *D.biguttulata*, *D.eres* and *D.unshiuensis*, were identified. In addition, twelve novel taxa were collected and are described as *D.acerigena*, *D.alangii*, *D.betulina*, *D.caryae*, *D.cercidis*, *D.chensiensis*, *D.cinnamomi*, *D.conica*, *D.fraxinicola*, *D.kadsurae*, *D.padina* and *D.ukurunduensis*. The current study improves the understanding of species causing diebacks on ecological and economic forest trees and provides useful information for the effective disease management of these hosts in China.

## Introduction

The genus *Diaporthe* Nitschke represents a cosmopolitan group of fungi occupying diverse ecological behaviour as plant pathogens, endophytes and saprobes (Muralli et al. 2006, Rossman et al. 2007, Garcia-Reyne et al. 2011, Udayanga et al. 2011, 2012a, b, 2014a, b, 2015, Gomes et al. 2013, Fan et al. 2015, Du et al. 2016, Dissanayake et al. 2017b, Guarnaccia and Crous 2017, Yang et al. 2017a, b, 2018, Guarnaccia et al. 2018, Marin-Felix et al. 2018). *Diaporthe* species are responsible for diseases on a wide range of plant hosts, including agricultural crops, forest trees and ornamentals, some of which are economically important. Several symptoms such as root and fruit rots, dieback, stem cankers, leaf spots, leaf and pod blights and seed decay are caused by *Diaporthe* spp. (Uecker 1988, Rehner and Uecker 1994, Mostert et al. 2001, Santos et al. 2011, Thompson et al. 2011, Udayanga et al. 2011). For example, *D.ampelina*, the causal agent of Phomopsis cane and leaf spot, is known as a severe pathogen of grapevines (Hewitt and Pearson 1988), infecting all green tissues and causing yield reductions of up to 30% in temperate regions (Erincik et al. 2001). *Diaporthecitri* is another well-known pathogen exclusively found on *Citrus* spp. causing melanose, stem-end rot and gummosis in all the citrus production areas except Europe (Mondal et al. 2007, Udayanga et al. 2014a, Guarnaccia and Crous 2017, 2018). Similarly, stem canker, attributed to several *Diaporthe* spp., is one of the most important diseases of sunflower (*Helianthusannuus*) worldwide (Muntañola-Cvetković et al. 1981, Thompson et al. 2011).

Several species of *Diaporthe* include a broad number of endophytes associated with hosts present in temperate and tropical regions (Udayanga et al. 2011). Gomes et al. (2013) considered that *D.endophytica* is a sterile endophyte on *Schinusterebinthifolius* and *Maytenusilicifolia* based on molecular phylogeny. Huang et al. (2015) distinguished seven undescribed *Diaporthe* species associated with citrus in China. Moreover, some endophytes have been shown to act as opportunistic plant pathogens. For instance, *D.foeniculina* has been found as both endophyte and opportunistic pathogen on various herbaceous weeds, ornamentals and fruit trees (Udayanga et al. 2014a, Guarnaccia et al. 2016).

The genus *Diaporthe* (syn. *Phomopsis*) was established by Nitschke (1870). Species identification criteria in *Diaporthe* were originally based on host association, morphology and culture characteristics (Mostert et al. 2001, Santos and Phillips 2009, Udayanga et al. 2012). As a consequence, a broad increase in the number of proposed *Diaporthe* species occurred. More than 1000 epithets for *Diaporthe* and 950 for *Phomopsis* were listed in Index Fungorum (2018) (http://www.indexfungorum.org/) (accessed 1 March 2018). The abolishment of the dual nomenclature system for pleomorphic fungi raised the question about which generic name to use. Given that both names are well known amongst plant pathologists and have been equally used, Rossman et al. (2015) proposed that the name *Diaporthe* (Nitschke 1870) has priority over *Phomopsis* (Saccardo and Roumeguère 1884) and has been adopted as the generic name in recent major studies (Gomes et al. 2013, Udayanga et al. 2014a, b, 2015, Fan et al. 2015, Huang et al. 2015, Du et al. 2016, Gao et al. 2017, Yang et al. 2017a, b, c, 2018).

The sexual morph of *Diaporthe* is characterised by immersed ascomata and an erumpent pseudostroma with elongated perithecial necks. Asci are unitunicate, clavate to cylindrical. Ascospores are fusoid, ellipsoid to cylindrical, hyaline, biseriate to uniseriate in the ascus and sometimes with appendages (Udayanga et al. 2011). The asexual morph is characterised by ostiolate conidiomata, with cylindrical phialides producing three types of hyaline, aseptate conidia (Udayanga et al. 2011). Previously, species identification of *Diaporthe* was largely referred to the assumption of host-specificity, leading to the proliferation of names (Gomes et al. 2013). More than one species of *Diaporthe* can colonise a single host, while one species can be associated with different hosts (Santos and Phillips 2009, Diogo et al. 2010, Santos et al. 2011, Gomes et al. 2013). In addition, considerable variability of the phenotype characters is present within a species (Rehner and Uecker 1994, Mostert et al. 2001, Santos et al. 2010, Udayanga et al. 2011, 2012a). Species identification is essential for understanding the epidemiology and plant diseases management and to guide the implementation of phytosanitary measures (Santos and Phillips 2009, Udayanga et al. 2011, Santos et al. 2017). Thus, molecular data are necessary to resolve *Diaporthe* taxonomy and, during the recent years, many species have been described through a polyphasic approach together with morphology (Gomes et al. 2013, Udayanga et al. 2014a, b, 2015, Huang et al. 2015, Gao et al. 2017, Guarnaccia and Crous 2017, Yang et al. 2018). Santos et al. (2017) revealed that the use of a five-loci dataset (ITS-*cal*-*his3*-*tef1*-*tub2*) is the optimal combination for species delimitation, showing the ribosomal ITS locus as the least informative, which is contrary to the result of Santos et al. (2010).

Although the classification of *Diaporthe* has been on-going, species are currently being identified based on a combination of morphological, cultural, phytopathological and phylogenetical analyses (Gomes et al. 2013, Huang et al. 2013, 2015, Udayanga et al. 2014a, b, 2015, Fan et al. 2015, Du et al. 2016, Gao et al. 2016, 2017, Guarnaccia and Crous 2017, Hyde et al. 2017, 2018, Guarnaccia et al. 2018, Jayawardena et al. 2018, Perera et al. 2018a, b, Tibpromma et al. 2018, Wanasinghe et al. 2018). However, fungi isolated from forest trees in China were recorded in old fungal literature without any living culture and molecular data (Teng 1963, Tai 1979, Wei 1979). The current study aimed to investigate the major ecological or economic trees in China by large-scale sampling and to identify isolates via morphology and multi-locus phylogeny based on modern taxonomic concepts. From 2015 to 2017, several surveys were conducted in six Provinces representing 16 host genera. The objectives of the present study were (i) to provide a multi-gene phylogeny for the genus *Diaporthe* based on a large set of freshly collected specimens in China; (ii) to identify *Diaporthe* taxa associated with disease symptoms or non-symptomatic tissues of various host genera distributed over six Provinces in China; (iii) to define the species limits of *D.eres* and closely related species based on multi-gene genealogies.

## Materials and methods

### Isolates

From 2015 to 2017, fresh specimens of *Diaporthe* were collected from symptomatic or non-symptomatic twigs or branches from Beijing, Heilongjiang, Jiangsu, Jiangxi, Shaanxi and Zhejiang Provinces in China (Table 1). A total of 105 isolates were obtained by removing a mucoid spore mass from conidiomata and spreading the suspension on the surface of 1.8% potato dextrose agar (PDA) in a Petri dish and incubating at 25 °C for up to 24 h. Single germinating conidia were transferred on to fresh PDA plates. Forty-two representative *Diaporthe* strains were selected based on cultural characteristics on PDA, conidia morphology and ITS sequence data. Specimens were deposited in the Museum of the Beijing Forestry University (BJFC). Axenic cultures are maintained in the China Forestry Culture Collection Centre (CFCC).

**Table 1.** Isolates and GenBank accession numbers used in the phylogenetic analyses of *Diaporthe*.

Species	Isolate	Host	Location	GenBank accession numbers				
				ITS	*cal*	*his3*	*tef1*	*tub2*
* D. acaciarum *	CBS 138862	* Acacia tortilis *	Tanzania	KP004460	N/A^a^	N/A^a^	N/A^a^	KP004509
* D. acaciigena *	CBS 129521	* Acacia retinodes *	Australia	KC343005	KC343247	KC343489	KC343731	KC343973
* D. acericola *	MFLUCC 17-0956	* Acer negundo *	Italy	KY964224	KY964137	N/A^a^	KY964180	KY964074
*** D. acerigena ***	**CFCC 52554**	*** Acer tataricum ***	**China**	**MH121489**	**MH121413**	**MH121449**	**MH121531**	**N/A^a^**
	**CFCC 52555**	*** Acer tataricum ***	**China**	**MH121490**	**MH121414**	**MH121450**	**MH121532**	**N/A^a^**
* D. acutispora *	CGMCC 3.18285	*Coffea* sp.	China	KX986764	KX999274	N/A^a^	KX999155	KX999195
*** D. alangii ***	**CFCC 52556**	*** Alangium kurzii ***	**China**	**MH121491**	**MH121415**	**MH121451**	**MH121533**	**MH121573**
	**CFCC 52557**	*** Alangium kurzii ***	**China**	**MH121492**	**MH121416**	**MH121452**	**MH121534**	**MH121574**
	**CFCC 52558**	*** Alangium kurzii ***	**China**	**MH121493**	**MH121417**	**MH121453**	**MH121535**	**MH121575**
	**CFCC 52559**	*** Alangium kurzii ***	**China**	**MH121494**	**MH121418**	**MH121454**	**MH121536**	**MH121576**
* D. alleghaniensis *	CBS 495.72	* Betula alleghaniensis *	Canada	KC343007	KC343249	KC343491	KC343733	KC343975
* D. alnea *	CBS 146.46	*Alnus* sp.	Netherlands	KC343008	KC343250	KC343492	KC343734	KC343976
* D. ambigua *	CBS 114015	* Pyrus communis *	South Africa	KC343010	KC343252	KC343494	KC343736	KC343978
* D. ampelina *	STEU2660	* Vitis vinifera *	France	AF230751	AY745026	N/A^a^	AY745056	JX275452
* D. amygdali *	CBS 126679	* Prunus dulcis *	Portugal	KC343022	KC343264	KC343506	AY343748	KC343990
* D. anacardii *	CBS 720.97	* Anacardium occidentale *	East Africa	KC343024	KC343266	KC343508	KC343750	KC343992
* D. angelicae *	CBS 111592	* Heracleum sphondylium *	Austria	KC343027	KC343269	KC343511	KC343753	KC343995
* D. apiculatum *	CGMCC 3.17533	* Camellia sinensis *	China	KP267896	N/A^a^	N/A^a^	KP267970	KP293476
* D. aquatica *	IFRDCC 3051	* Aquatic habitat *	China	JQ797437	N/A^a^	N/A^a^	N/A^a^	N/A^a^
* D. arctii *	CBS 139280	* Arctium lappa *	Austria	KJ590736	KJ612133	KJ659218	KJ590776	KJ610891
* D. arecae *	CBS 161.64	* Areca catechu *	India	KC343032	KC343274	KC343516	KC343758	KC344000
* D. arengae *	CBS 114979	* Arenga enngleri *	Hong Kong	KC343034	KC343276	KC343518	KC343760	KC344002
* D. aseana *	MFLUCC 12-0299a	Unknown dead leaf	Thailand	KT459414	KT459464	N/A^a^	KT459448	KT459432
* D. asheicola *	CBS 136967	* Vaccinium ashei *	Chile	KJ160562	KJ160542	N/Aa	KJ160594	KJ160518
* D. aspalathi *	CBS 117169	* Aspalathus linearis *	South Africa	KC343036	KC343278	KC343520	KC343762	KC344004
* D. australafricana *	CBS 111886	* Vitis vinifera *	Australia	KC343038	KC343280	KC343522	KC343764	KC344006
* D. baccae *	CBS 136972	* Vaccinium corymbosum *	Italy	KJ160565	N/A^a^	MF418264	KJ160597	N/A^a^
* D. batatas *	CBS 122.21	* Ipomoea batatas *	USA	KC343040	KC343282	N/A^a^	KC343766	KC344008
* D. beilharziae *	BRIP 54792	* Indigofera australis *	Australia	JX862529	N/A^a^	N/A^a^	JX862535	KF170921
* D. benedicti *	BPI 893190	*Salix* sp.	USA	KM669929	KM669862	N/A^a^	KM669785	N/A^a^
* D. betulae *	CFCC 50469	* Betula platyphylla *	China	KT732950	KT732997	KT732999	KT733016	KT733020
	CFCC 50470	* Betula platyphylla *	China	KT732951	KT732998	KT733000	KT733017	KT733021
* D. betulicola *	CFCC 51128	* Betula albo-sinensis *	China	KX024653	KX024659	KX024661	KX024655	KX024657
	CFCC 51129	* Betula albo-sinensis *	China	KX024654	KX024660	KX024662	KX024656	KX024658
*** D. betulina ***	**CFCC 52560**	*** Betula albo-sinensis ***	**China**	**MH121495**	**MH121419**	**MH121455**	**MH121537**	**MH121577**
	**CFCC 52561**	*** Betula costata ***	**China**	**MH121496**	**MH121420**	**MH121456**	**MH121538**	**MH121578**
	**CFCC 52562**	*** Betula platyphylla ***	**China**	**MH121497**	**MH121421**	**MH121457**	**MH121539**	**MH121579**
* D. bicincta *	CBS 121004	*Juglans* sp.	USA	KC343134	KC343376	KC343618	KC343860	KC344102
* D. biconispora *	CGMCC 3.17252	* Citrus grandis *	China	KJ490597	KJ490539	KJ490539	KJ490476	KJ490418
* D. biguttulata *	CGMCC 3.17248	* Citrus limon *	China	KJ490582	N/A^a^	KJ490524	KJ490461	KJ490403
	**CFCC 52584**	*** Juglans regia ***	**China**	**MH121519**	**MH121437**	**MH121477**	**MH121561**	**MH121598**
	**CFCC 52585**	*** Juglans regia ***	**China**	**MH121520**	**MH121438**	**MH121478**	**MH121562**	**MH121599**
* D. biguttusis *	CGMCC 3.17081	* Lithocarpus glabra *	China	KF576282	N/A^a^	N/A^a^	KF576257	KF576306
* D. bohemiae *	CPC 28222	* Vitis vinifera *	Czech Republic	MG281015	MG281710	MG281361	MG281536	MG281188
* D. brasiliensis *	CBS 133183	* Aspidosperma tomentosum *	Brazil	KC343042	KC343284	KC343526	KC343768	KC344010
* D. caatingaensis *	CBS 141542	* Tacinga inamoena *	Brazil	KY085927	N/A^a^	N/A^a^	KY115603	KY115600
* D. camptothecicola *	CFCC 51632	* Camptotheca acuminata *	China	KY203726	KY228877	KY228881	KY228887	KY228893
* D. canthii *	CBS 132533	* Canthium inerme *	South Africa	JX069864	KC843174	N/A^a^	KC843120	KC843230
*** D. caryae ***	**CFCC 52563**	*** Carya illinoensis ***	**China**	**MH121498**	**MH121422**	**MH121458**	**MH121540**	**MH121580**
	**CFCC 52564**	*** Carya illinoensis ***	**China**	**MH121499**	**MH121423**	**MH121459**	**MH121541**	**MH121581**
* D. cassines *	CPC 21916	* Cassine peragua *	South Africa	KF777155	N/A^a^	N/A^a^	KF777244	N/A^a^
* D. caulivora *	CBS 127268	* Glycine max *	Croatia	KC343045	KC343287	N/A^a^	KC343771	KC344013
* D. celeris *	CPC 28262	* Vitis vinifera *	Czech Republic	MG281017	MG281712	MG281363	MG281538	MG281190
* D. celastrina *	CBS 139.27	*Celastrus* sp.	USA	KC343047	KC343289	KC343531	KC343773	KC344015
*** D. cercidis ***	**CFCC 52565**	*** Cercis chinensis ***	**China**	**MH121500**	**MH121424**	**MH121460**	**MH121542**	**MH121582**
	**CFCC 52566**	*** Cercis chinensis ***	**China**	**MH121501**	**MH121425**	**MH121461**	**MH121543**	**MH121583**
* D. chamaeropis *	CBS 454.81	* Chamaerops humilis *	Greece	KC343048	KC343290	KC343532	KC343774	KC344016
* D. charlesworthii *	BRIP 54884m	* Rapistrum rugostrum *	Australia	KJ197288	N/A^a^	N/A^a^	KJ197250	KJ197268
*** D. chensiensis ***	**CFCC 52567**	*** Abies chensiensis ***	**China**	**MH121502**	**MH121426**	**MH121462**	**MH121544**	**MH121584**
	**CFCC 52568**	*** Abies chensiensis ***	**China**	**MH121503**	**MH121427**	**MH121463**	**MH121545**	**MH121585**
* D. cichorii *	MFLUCC 17-1023	* Cichorium intybus *	Italy	KY964220	KY964133	N/A^a^	KY964176	KY964104
*** D. cinnamomi ***	**CFCC 52569**	***Cinnamomum* sp.**	**China**	**MH121504**	N/A^a^	**MH121464**	**MH121546**	**MH121586**
	**CFCC 52570**	***Cinnamomum* sp.**	**China**	**MH121505**	N/A^a^	**MH121465**	**MH121547**	**MH121587**
* D. cissampeli *	CBS 141331	* Cissampelos capensis *	South Africa	KX228273	N/A^a^	KX228366	N/A^a^	KX228384
* D. citri *	AR 3405	*Citrus* sp.	USA	KC843311	KC843157	N/A^a^	KC843071	KC843187
* D. citriasiana *	CGMCC 3.15224	* Citrus unshiu *	China	JQ954645	KC357491	KJ490515	JQ954663	KC357459
*D.citrichinensi*s	CGMCC 3.15225	*Citrus* sp.	China	JQ954648	KC357494	N/A^a^	JQ954666	N/A^a^
* D. collariana *	MFLU 17-2770	* Magnolia champaca *	Thailand	MG806115	MG783042	N/A^a^	MG783040	MG783041
* D. compacta *	CGMCC 3.17536	* Camellia sinensis *	China	KP267854	N/A^a^	KP293508	KP267928	KP293434
*** D. conica ***	**CFCC 52571**	*** Alangium chinense ***	**China**	**MH121506**	**MH121428**	**MH121466**	**MH121548**	**MH121588**
	**CFCC 52572**	*** Alangium chinense ***	**China**	**MH121507**	**MH121429**	**MH121467**	**MH121549**	**MH121589**
	**CFCC 52573**	*** Alangium chinense ***	**China**	**MH121508**	**MH121430**	**MH121468**	**MH121550**	**MH121590**
	**CFCC 52574**	*** Alangium chinense ***	**China**	**MH121509**	**MH121431**	**MH121469**	**MH121551**	**MH121591**
* D. convolvuli *	CBS 124654	* Convolvulus arvensis *	Turkey	KC343054	KC343296	KC343538	KC343780	KC344022
* D. crotalariae *	CBS 162.33	* Crotalaria spectabilis *	USA	KC343056	KC343298	KC343540	KC343782	KC344024
* D. cucurbitae *	CBS 136.25	*Arctium* sp.	Unknown	KC343031	KC343273	KC343515	KC343757	KC343999
* D. cuppatea *	CBS 117499	* Aspalathus linearis *	South Africa	KC343057	KC343299	KC343541	KC343783	KC344025
* D. cynaroidis *	CBS 122676	* Protea cynaroides *	South Africa	KC343058	KC343300	KC343542	KC343784	KC344026
* D. cytosporella *	FAU461	* Citrus limon *	Italy	KC843307	KC843141	N/A^a^	KC843116	KC843221
* D. diospyricola *	CPC 21169	* Diospyros whyteana *	South Africa	KF777156	N/A^a^	N/A^a^	N/A^a^	N/A^a^
* D. discoidispora *	ZJUD89	* Citrus unshiu *	China	KJ490624	N/A^a^	KJ490566	KJ490503	KJ490445
* D. dorycnii *	MFLUCC 17-1015	* Dorycnium hirsutum *	Italy	KY964215	N/A^a^	N/A^a^	KY964171	KY964099
* D. elaeagni-glabrae *	CGMCC 3.18287	* Elaeagnus glabra *	China	KX986779	KX999281	KX999251	KX999171	KX999212
* D. ellipicola *	CGMCC 3.17084	* Lithocarpus glabra *	China	KF576270	N/A^a^	N/A^a^	KF576245	KF576291
* D. endophytica *	CBS 133811	* Schinus terebinthifolius *	Brazil	KC343065	KC343307	KC343549	KC343791	KC343065
*** D. eres ***	AR5193	*Ulmus* sp.	Germany	KJ210529	KJ434999	KJ420850	KJ210550	KJ420799
	**CFCC 52575**	*** Castanea mollissima ***	**China**	**MH121510**	**N/A^a^**	**MH121470**	**MH121552**	**MH121592**
	**CFCC 52576**	*** Castanea mollissima ***	**China**	**MH121511**	**MH121432**	**MH121471**	**MH121553**	**MH121593**
	**CFCC 52577**	*** Acanthopanax senticosus ***	**China**	**MH121512**	**MH121433**	**MH121472**	**MH121554**	**MH121594**
	**CFCC 52578**	***Sorbus* sp.**	**China**	**MH121513**	**MH121434**	**MH121473**	**MH121555**	**MH121595**
	**CFCC 52579**	*** Juglans regia ***	**China**	**MH121514**	**N/A^a^**	**MH121474**	**MH121556**	**N/A^a^**
	**CFCC 52580**	*** Melia azedarace ***	**China**	**MH121515**	**N/A^a^**	**MH121475**	**MH121557**	**MH121596**
	**CFCC 52581**	*** Rhododendron simsii ***	**China**	**MH121516**	**N/A^a^**	**MH121476**	**MH121558**	**MH121597**
* D. eucalyptorum *	CBS 132525	*Eucalyptus* sp.	Australia	NR120157	N/Aa	N/Aa	N/Aa	N/Aa
* D. foeniculacea *	CBS 123208	* Foeniculum vulgare *	Portugal	KC343104	KC343346	KC343588	KC343830	KC344072
* D. fraxini-angustifoliae *	BRIP 54781	* Fraxinus angustifolia *	Australia	JX862528	N/A^a^	N/A^a^	JX862534	KF170920
*** D. fraxinicola ***	**CFCC 52582**	*** Fraxinus chinensis ***	**China**	**MH121517**	**MH121435**	**N/A^a^**	**MH121559**	**N/A^a^**
	**CFCC 52583**	*** Fraxinus chinensis ***	**China**	**MH121518**	**MH121436**	**N/A^a^**	**MH121560**	**N/A^a^**
* D. fukushii *	MAFF 625034	* Pyrus pyrifolia *	Japan	JQ807469	N/A^a^	N/A^a^	JQ807418	N/A^a^
* D. fusicola *	CGMCC 3.17087	* Lithocarpus glabra *	China	KF576281	KF576233	N/A^a^	KF576256	KF576305
* D. ganjae *	CBS 180.91	* Cannabis sativa *	USA	KC343112	KC343354	KC343596	KC343838	KC344080
* D. garethjonesii *	MFLUCC 12-0542a	*Unknown dead leaf*	Thailand	KT459423	KT459470	N/A^a^	KT459457	KT459441
* D. goulteri *	BRIP 55657a	* Helianthus annuus *	Australia	KJ197290	N/A^a^	N/A^a^	KJ197252	KJ197270
* D. gulyae *	BRIP 54025	* Helianthus annuus *	Australia	JF431299	N/A^a^	N/A^a^	KJ197271	JN645803
* D. helianthi *	CBS 592.81	* Helianthus annuus *	Serbia	KC343115	KC343357	KC343599	KC343841	KC344083
* D. helicis *	AR5211	* Hedera helix *	France	KJ210538	KJ435043	KJ420875	KJ210559	KJ420828
* D. heterophyllae *	CBS 143769	* Acacia heterohpylla *	France	MG600222	MG600218	MG600220	MG600224	MG600226
* D. hickoriae *	CBS 145.26	* Carya glabra *	USA	KC343118	KC343360	KC343602	KC343844	KC344086
* D. hispaniae *	CPC 30321	* Vitis vinifera *	Spain	MG281123	MG281820	MG281471	MG281644	MG281296
* D. hongkongensis *	CBS 115448	*Dichroa febrífuga*	China	KC343119	KC343361	KC343603	KC343845	KC344087
* D. incompleta *	CGMCC 3.18288	* Camellia sinensis *	China	KX986794	KX999289	KX999265	KX999186	KX999226
* D. inconspicua *	CBS 133813	* Maytenus ilicifolia *	Brazil	KC343123	KC343365	KC343607	KC343849	KC344091
* D. infecunda *	CBS 133812	* Schinus terebinthifolius *	Brazil	KC343126	KC343368	KC343610	KC343852	KC344094
* D. isoberliniae *	CPC 22549	* Isoberlinia angolensis *	Zambia	KJ869133	N/A^a^	N/A^a^	N/A^a^	KJ869245
* D. juglandicola *	CFCC 51134	* Juglans mandshurica *	China	KU985101	KX024616	KX024622	KX024628	KX024634
	CFCC 51135	* Juglans mandshurica *	China	KU985102	KX024617	KX024623	KX024629	KX024635
*** D. kadsurae ***	**CFCC 52586**	*** Kadsura longipedunculata ***	**China**	**MH121521**	**MH121439**	**MH121479**	**MH121563**	**MH121600**
	**CFCC 52587**	*** Kadsura longipedunculata ***	**China**	**MH121522**	**MH121440**	**MH121480**	**MH121564**	**MH121601**
	**CFCC 52588**	***Acer* sp.**	**China**	**MH121523**	**MH121441**	**MH121481**	**MH121565**	**MH121602**
	**CFCC 52589**	***Acer* sp.**	**China**	**MH121524**	**MH121442**	**MH121482**	**MH121566**	**MH121603**
* D. kochmanii *	BRIP 54033	* Helianthus annuus *	Australia	JF431295	N/A^a^	N/A^a^	JN645809	N/A^a^
* D. kongii *	BRIP 54031	* Portulaca grandiflora *	Australia	JF431301	N/A^a^	N/A^a^	JN645797	KJ197272
* D. litchicola *	BRIP 54900	* Litchi chinensis *	Australia	JX862533	N/A^a^	N/A^a^	JX862539	KF170925
* D. lithocarpus *	CGMCC 3.15175	* Lithocarpus glabra *	China	KC153104	KF576235	N/A^a^	KC153095	KF576311
* D. longicicola *	CGMCC 3.17089	* Lithocarpus glabra *	China	KF576267	N/A^a^	N/A^a^	KF576242	KF576291
* D. longicolla *	ATCC 60325	* Glycine max *	USA	KJ590728	N/A^a^	KJ659188	KJ590767	KJ610883
* D. longispora *	CBS 194.36	*Ribes* sp.	Canada	KC343135	KC343377	KC343619	KC343861	KC344103
* D. lonicerae *	MFLUCC 17-0963	*Lonicera* sp.	Italy	KY964190	KY964116	N/A^a^	KY964146	KY964073
* D. lusitanicae *	CBS 123212	* Foeniculum vulgare *	Portugal	KC343136	KC343378	KC343620	KC343862	KC344104
* D. macinthoshii *	BRIP 55064a	* Rapistrum rugostrum *	Australia	KJ197289	N/A^a^	N/A^a^	KJ197251	KJ197269
* D. mahothocarpus *	CGMCC 3.15181	* Lithocarpus glabra *	China	KC153096	N/A^a^	N/A^a^	KC153087	KF576312
* D. malorum *	CAA734	* Malus domestica *	Portugal	KY435638	KY435658	KY435648	KY435627	KY435668
* D. maritima *	DAOMC 250563	* Picea rubens *	Canada	N/A^a^	N/A^a^	N/A^a^	N/A^a^	KU574616
* D. masirevicii *	BRIP 57892a	* Helianthus annuus *	Australia	KJ197277	N/A^a^	N/A^a^	KJ197239	KJ197257
* D. mayteni *	CBS 133185	* Maytenus ilicifolia *	Brazil	KC343139	KC343381	KC343623	KC343865	KC344107
* D. maytenicola *	CPC 21896*	* Maytenus acuminata *	South Africa	KF777157	N/A^a^	N/A^a^	N/A^a^	KF777250
* D. melonis *	CBS 507.78	* Cucumis melo *	USA	KC343142	KC343384	KC343626	KC343868	KC344110
* D. middletonii *	BRIP 54884e	* Rapistrum rugostrum *	Australia	KJ197286	N/A^a^	N/A^a^	KJ197248	KJ197266
* D. miriciae *	BRIP 54736j	* Helianthus annuus *	Australia	KJ197282	N/A^a^	N/A^a^	KJ197244	KJ197262
* D. momicola *	MFLUCC 16-0113	* Prunus persica *	China	KU557563	KU557611	N/A^a^	KU557631	KU55758
* D. multigutullata *	ZJUD98	* Citrus grandis *	China	KJ490633	N/A^a^	KJ490575	KJ490512	KJ490454
* D. musigena *	CBS 129519	*Musa* sp.	Australia	KC343143	KC343385	KC343627	KC343869	KC344111
* D. neilliae *	CBS 144.27	*Spiraea* sp.	USA	KC343144	KC343386	KC343628	KC343870	KC344112
* D. neoarctii *	CBS 109490	* Ambrosia trifida *	USA	KC343145	KC343387	KC343629	KC343871	KC344113
* D. neoraonikayaporum *	MFLUCC 14-1136	* Tectona grandis *	Thailand	KU712449	KU749356	N/A^a^	KU749369	KU743988
* D. nobilis *	CBS 113470	* Castanea sativa *	Korea	KC343146	KC343388	KC343630	KC343872	KC344114
* D. nothofagi *	BRIP 54801	* Nothofagus cunninghamii *	Australia	JX862530	N/A^a^	N/A^a^	JX862536	KF170922
* D. novem *	CBS 127270	* Glycine max *	Croatia	KC343155	KC343397	KC343640	KC343881	KC344123
* D. ocoteae *	CBS 141330	* Ocotea obtusata *	France	KX228293	N/A^a^	N/A^a^	N/A^a^	KX228388
* D. oraccinii *	CGMCC 3.17531	* Camellia sinensis *	China	KP267863	N/A^a^	KP293517	KP267937	KP293443
* D. ovalispora *	ICMP20659	* Citrus limon *	China	KJ490628	N/A^a^	KJ490570	KJ490507	KJ490449
* D. ovoicicola *	CGMCC 3.17093	*Citrus* sp.	China	KF576265	KF576223	N/A^a^	KF576240	KF576289
* D. oxe *	CBS 133186	* Maytenus ilicifolia *	Brazil	KC343164	KC343406	KC343648	KC343890	KC344132
*** D. padina ***	**CFCC 52590**	*** Padus racemosa ***	**China**	**MH121525**	**MH121443**	**MH121483**	**MH121567**	**MH121604**
	**CFCC 52591**	*** Padus racemosa ***	**China**	**MH121526**	**MH121444**	**MH121484**	**MH121568**	**MH121605**
* D. pandanicola *	MFLU 18-0006	*Pandanus* sp.	Thailand	MG646974	N/A^a^	N/A^a^	N/A^a^	MG646930
* D. paranensis *	CBS 133184	* Maytenus ilicifolia *	Brazil	KC343171	KC343413	KC343655	KC343897	KC344139
* D. parapterocarpi *	CPC 22729	* Pterocarpus brenanii *	Zambia	KJ869138	N/A^a^	N/A^a^	N/A^a^	KJ869248
* D. pascoei *	BRIP 54847	* Persea americana *	Australia	JX862532	N/A^a^	N/A^a^	JX862538	KF170924
* D. passiflorae *	CBS 132527	* Passiflora edulis *	South America	JX069860	N/A^a^	KY435654	N/A^a^	N/A^a^
* D. passifloricola *	CBS 141329	* Passiflora foetida *	Malaysia	KX228292	N/A^a^	KX228367	N/A^a^	KX228387
* D. penetriteum *	CGMCC 3.17532	* Camellia sinensis *	China	KP714505	N/A^a^	KP714493	KP714517	KP714529
* D. perjuncta *	CBS 109745	* Ulmus glabra *	Austria	KC343172	KC343414	KC343656	KC343898	KC344140
* D. perseae *	CBS 151.73	* Persea gratissima *	Netherlands	KC343173	KC343415	KC343657	KC343899	KC344141
* D. pescicola *	MFLUCC 16-0105	* Prunus persica *	China	KU557555	KU557603	N/A^a^	KU557623	KU557579
* D. phaseolorum *	AR4203	* Phaseolus vulgaris *	USA	KJ590738	N/A^a^	KJ659220	N/A^a^	KP004507
* D. podocarpi-macrophylli *	CGMCC 3.18281	* Podocarpus macrophyllus *	China	KX986774	KX999278	KX999246	KX999167	KX999207
* D. pseudomangiferae *	CBS 101339	* Mangifera indica *	Dominican Republic	KC343181	KC343423	KC343665	KC343907	KC344149
* D. pseudophoenicicola *	CBS 462.69	* Phoenix dactylifera *	Spain	KC343184	KC343426	KC343668	KC343910	KC344152
* D. pseudotsugae *	MFLU 15-3228	* Pseudotsuga menziesii *	Italy	KY964225	KY964138	N/A^a^	KY964181	KY964108
* D. psoraleae *	CBS 136412	* Psoralea pinnata *	South Africa	KF777158	N/A^a^	N/A^a^	KF777245	KF777251
* D. psoraleae-pinnatae *	CBS 136413	* Psoralea pinnata *	South Africa	KF777159	N/A^a^	N/A^a^	N/A^a^	KF777252
* D. pterocarpi *	MFLUCC 10-0571	* Pterocarpus indicus *	Thailand	JQ619899	JX197451	N/A^a^	JX275416	JX275460
* D. pterocarpicola *	MFLUCC 10-0580a	* Pterocarpus indicus *	Thailand	JQ619887	JX197433	N/A^a^	JX275403	JX275441
* D. pulla *	CBS 338.89	* Hedera helix *	Yugoslavia	KC343152	KC343394	KC343636	KC343878	KC344120
* D. pyracanthae *	CAA483	* Pyracantha coccinea *	Portugal	KY435635	KY435656	KY435645	KY435625	KY435666
* D. racemosae *	CBS 143770	* Euclea racemosa *	South Africa	MG600223	MG600219	MG600221	MG600225	MG600227
* D. raonikayaporum *	CBS 133182	* Spondias mombin *	Brazil	KC343188	KC343430	KC343672	KC343914	KC344156
* D. ravennica *	MFLUCC 15-0479	*Tamarix* sp.	Italy	KU900335	N/A^a^	N/A^a^	KX365197	KX432254
* D. rhusicola *	CBS 129528	* Rhus pendulina *	South Africa	JF951146	KC843124	N/A^a^	KC843100	KC843205
* D. rosae *	MFLU 17-1550	*Rosa* sp.	Thailand	MG828894	N/A^a^	N/A^a^	N/A^a^	MG843878
* D. rosicola *	MFLU 17-0646	*Rosa* sp.	UK	MG828895	N/A^a^	N/A^a^	MG829270	MG843877
* D. rostrata *	CFCC 50062	* Juglans mandshurica *	China	KP208847	KP208849	KP208851	KP208853	KP208855
	CFCC 50063	* Juglans mandshurica *	China	KP208848	KP208850	KP208852	KP208854	KP208856
* D. rudis *	AR3422	* Laburnum anagyroides *	Austria	KC843331	KC843146	N/A^a^	KC843090	KC843177
* D. saccarata *	CBS 116311	* Protea repens *	South Africa	KC343190	KC343432	KC343674	KC343916	KC344158
* D. sackstonii *	BRIP 54669b	* Helianthus annuus *	Australia	KJ197287	N/A^a^	N/A^a^	KJ197249	KJ197267
* D. salicicola *	BRIP 54825	* Salix purpurea *	Australia	JX862531	N/A^a^	N/A^a^	JX862537	JX862531
* D. sambucusii *	CFCC 51986	* Sambucus williamsii *	China	KY852495	KY852499	KY852503	KY852507	KY852511
	CFCC 51987	* Sambucus williamsii *	China	KY852496	KY852500	KY852504	KY852508	KY852512
* D. schini *	CBS 133181	* Schinus terebinthifolius *	Brazil	KC343191	KC343433	KC343675	KC343917	KC344159
* D. schisandrae *	CFCC 51988	* Schisandra chinensis *	China	KY852497	KY852501	KY852505	KY852509	KY852513
	CFCC 51989	* Schisandra chinensis *	China	KY852498	KY852502	KY852506	KY852510	KY852514
* D. schoeni *	MFLU 15-1279	* Schoenus nigricans *	Italy	KY964226	KY964139	N/A^a^	KY964182	KY964109
* D. sclerotioides *	CBS 296.67	* Cucumis sativus *	Netherlands	KC343193	KC343435	KC343677	KC343919	KC344161
* D. sennae *	CFCC 51636	* Senna bicapsularis *	China	KY203724	KY228875	N/A^a^	KY228885	KY228891
	CFCC 51637	* Senna bicapsularis *	China	KY203725	KY228876	N/Aa	KY228886	KY228892
* D. sennicola *	CFCC 51634	* Senna bicapsularis *	China	KY203722	KY228873	KY228879	KY228883	KY228889
	CFCC 51635	* Senna bicapsularis *	China	KY203723	KY228874	KY228880	KY228884	KY228890
* D. serafiniae *	BRIP 55665a	* Helianthus annuus *	Australia	KJ197274	N/A^a^	N/A^a^	KJ197236	KJ197254
* D. siamensis *	MFLUCC 10-573a	*Dasymaschalon* sp.	Thailand	JQ619879	N/A^a^	N/A^a^	JX275393	JX275429
* D. sojae *	FAU635	* Glycine max *	USA	KJ590719	KJ612116	KJ659208	KJ590762	KJ610875
* D. spartinicola *	CBS 140003	* Spartium junceum *	Spain	KR611879	N/A^a^	KR857696	N/A^a^	KR857695
* D. sterilis *	CBS 136969	* Vaccinium corymbosum *	Italy	KJ160579	KJ160548	MF418350	KJ160611	KJ160528
* D. stictica *	CBS 370.54	* Buxus sampervirens *	Italy	KC343212	KC343454	KC343696	KC343938	KC344180
* D. subclavata *	ICMP20663	* Citrus unshiu *	China	KJ490587	N/A^a^	KJ490529	KJ490466	KJ490408
* D. subcylindrospora *	MFLU 17-1195	*Salix* sp.	China	MG746629	N/A^a^	N/A^a^	MG746630	MG746631
* D. subellipicola *	MFLU 17-1197	on dead wood	China	MG746632	N/A^a^	N/A^a^	MG746633	MG746634
* D. subordinaria *	CBS 464.90	* Plantago lanceolata *	New Zealand	KC343214	KC343456	KC343698	KC343940	KC344182
* D. taoicola *	MFLUCC 16-0117	* Prunus persica *	China	KU557567	N/Aa	N/A^a^	KU557635	KU557591
* D. tectonae *	MFLUCC 12-0777	* Tectona grandis *	China	KU712430	KU749345	N/A^a^	KU749359	KU743977
* D. tectonendophytica *	MFLUCC 13-0471	* Tectona grandis *	China	KU712439	KU749354	N/A^a^	KU749367	KU749354
* D. tectonigena *	MFLUCC 12-0767	* Tectona grandis *	China	KU712429	KU749358	N/A^a^	KU749371	KU743976
* D. terebinthifolii *	CBS 133180	* Schinus terebinthifolius *	Brazil	KC343216	KC343458	KC343700	KC343942	KC344184
* D. thunbergii *	MFLUCC 10-576a	* Thunbergia laurifolia *	Thailand	JQ619893	JX197440	N/A^a^	JX275409	JX275449
* D. thunbergiicola *	MFLUCC 12-0033	* Thunbergia laurifolia *	Thailand	KP715097	N/A^a^	N/A^a^	KP715098	N/A^a^
* D. tibetensis *	CFCC 51999	* Juglandis regia *	China	MF279843	MF279888	MF279828	MF279858	MF279873
	CFCC 52000	* Juglandis regia *	China	MF279844	MF279889	MF279829	MF279859	MF279874
* D. torilicola *	MFLUCC 17-1051	* Torilis arvensis *	Italy	KY964212	KY964127	N/A^a^	KY964168	KY964096
* D. toxica *	CBS 534.93	* Lupinus angustifolius *	Australia	KC343220	KC343462	C343704	KC343946	KC344188
* D. tulliensis *	BRIP 62248a	*Theobromacacao* fruit	Australia	KR936130	N/A^a^	N/A^a^	KR936133	KR936132
* D. ueckerae *	FAU656	* Cucumis melo *	USA	KJ590726	KJ612122	KJ659215	KJ590747	KJ610881
*** D. ukurunduensis ***	**CFCC 52592**	*** Acer ukurunduense ***	**China**	**MH121527**	**MH121445**	**MH121485**	**MH121569**	N/A^a^
	**CFCC 52593**	*** Acer ukurunduense ***	**China**	**MH121528**	**MH121446**	**MH121486**	**MH121570**	N/A^a^
* D. undulata *	CGMCC 3.18293	Leaf of unknown host	China-Laos border	KX986798	N/A^a^	KX999269	KX999190	KX999230
*** D. unshiuensis ***	CGMCC 3.17569	* Citrus unshiu *	China	KJ490587	N/A^a^	KJ490529	KJ490408	KJ490466
	**CFCC 52594**	*** Carya illinoensis ***	**China**	**MH121529**	**MH121447**	**MH121487**	**MH121571**	**MH121606**
	**CFCC 52595**	*** Carya illinoensis ***	**China**	**MH121530**	**MH121448**	**MH121488**	**MH121572**	**MH121607**
* D. vaccinii *	CBS 160.32	* Oxycoccus macrocarpos *	USA	KC343228	KC343470	KC343712	KC343954	KC344196
* D. vangueriae *	CPC 22703	* Vangueria infausta *	Zambia	KJ869137	N/A^a^	N/A^a^	N/A^a^	KJ869247
* D. vawdreyi *	BRIP 57887a	* Psidium guajava *	Australia	KR936126	N/A^a^	N/A^a^	KR936129	KR936128
* D. velutina *	CGMCC 3.18286	*Neolitsea* sp.	China	KX986790	N/A^a^	KX999261	KX999182	KX999223
* D. virgiliae *	CMW40748	* Virgilia oroboides *	South Africa	KP247566	N/A^a^	N/A^a^	N/A^a^	KP247575
* D. xishuangbanica *	CGMCC 3.18282	* Camellia sinensis *	China	KX986783	N/A^a^	KX999255	KX999175	KX999216
* D. yunnanensis *	CGMCC 3.18289	*Coffea* sp.	China	KX986796	KX999290	KX999267	KX999188	KX999228
* Diaporthella corylina *	CBS 121124	*Corylus* sp.	China	KC343004	KC343246	KC343488	KC343730	KC343972

Newly sequenced material is indicated in bold type.

### Morphological analysis

Agar plugs (6 mm diam.) were taken from the edge of actively growing cultures on PDA and transferred on to the centre of 9 cm diam Petri dishes containing 2% tap water agar supplemented with sterile pine needles (PNA; Smith et al. 1996) and potato dextrose agar (PDA) and incubated at 20–21 °C under a 12 h near-ultraviolet light/12 h dark cycle to induce sporulation as described in recent studies (Gomes et al. 2013, Lombard et al. 2014). Colony characters and pigment production on PNA and PDA were noted after 10 d. Colony colours were rated according to Rayner (1970). Cultures were examined periodically for the development of ascomata and conidiomata. The morphological characteristics were examined by mounting fungal structures in clear lactic acid and 30 measurements at 1000× magnification were determined for each isolate using a Leica compound microscope (DM 2500) with interference contrast (DIC) optics. Descriptions, nomenclature and illustrations of taxonomic novelties are deposited in MycoBank (www.MycoBank.org; Crous et al. 2004b).

### DNA extraction, PCR amplification and sequencing

Genomic DNA was extracted from colonies grown on cellophane-covered PDA using a modified CTAB [cetyltrimethylammonium bromide] method (Doyle and Doyle 1990). DNA was estimated by electrophoresis in 1% agarose gel and the quality was measured using the NanoDrop 2000 (Thermo Scientific, Waltham, MA, USA), following the user manual (Desjardins et al. 2009). PCR amplifications were performed in a DNA Engine Peltier Thermal Cycler (PTC-200; Bio-Rad Laboratories, Hercules, CA, USA). The primer sets ITS1/ITS4 (White et al. 1990) were used to amplify the ITS region. The primer pair CAL228F/CAL737R (Carbone and Kohn 1999) were used to amplify the calmodulin gene (*cal*) and the primer pair CYLH4F (Crous et al. 2004a) and H3-1b (Glass and Donaldson 1995) were used to amplify part of the histone H3 (*his3*) gene. The primer pair EF1-728F/EF1-986R (Carbone and Kohn 1999) were used to amplify a partial fragment of the translation elongation factor 1-α gene (*tef1*). The primer sets T1 (O’Donnell and Cigelnik 1997) and Bt2b (Glass and Donaldson 1995) were used to amplify the beta-tubulin gene (*tub2*); the additional combination of Bt2a/Bt2b (Glass and Donaldson 1995) was used in case of amplification failure of the T1/Bt2b primer pair. Amplifications of different loci were performed under different conditions (Table 2). PCR amplification products were assayed via electrophoresis in 2% agarose gels. DNA sequencing was performed using an ABI PRISM® 3730XL DNA Analyser with a BigDye Terminater Kit v.3.1 (Invitrogen, USA) at the Shanghai Invitrogen Biological Technology Company Limited (Beijing, China).

**Table 2.** Genes used in this study with PCR primers, process and references.

Gene	PCR primers (forward/reverse)	PCR: thermal cycles: (Annealing temp. in bold)	References of primers used
ITS	ITS1/ITS4	(95 °C: 30 s, **51 °C**: 30 s, 72 °C: 1 min) × 35 cycles	White et al. 1990
*cal*	CAL228F/CAL737R	(95 °C: 15 s, **55 °C**: 20 s, 72 °C: 1 min) × 35 cycles	Carbone and Kohn 1999
*his3*	CYLH4F/H3-1b	(95 °C: 30 s, **58** °C: 30 s, 72 °C: 1 min) × 35 cycles	Glass and Donaldson 1995, Crous et al. 2004a
*tef1*	EF1-728F/EF1-986R	(9 °C: 15 s, **55 °C**: 20 s, 72 °C: 1 min) × 35 cycles	Carbone and Kohn 1999
*tub2*	T1(Bt2a)/Bt2b	(95 °C: 30 s, **55** °C: 30 s, 72 °C: 1 min) × 35 cycles	Glass and Donaldson 1995, Glass and Donaldson 1995

### Phylogenetic analyses

DNA generated sequences were used to obtain consensus sequences using SeqMan v.7.1.0 DNASTAR Lasergene Core Suite software programme (DNASTAR Inc., Madison, WI, USA). Sequences were aligned using MAFFT v.6 (Katoh and Toh 2010) and edited manually using MEGA6 (Tamura et al. 2013). Two different datasets were employed to estimate two phylogenetic analyses: one for *Diaporthe* species and one for *Diaportheeres* complex. The first analysis was undertaken to infer the interspecific relationships in *Diaporthe*. All the *Diaporthe* isolates recovered from samples collected during this study and additional reference sequences of *Diaporthe* species were included in the dataset of combined ITS, *cal*, *his3*, *tef1*, and *tub2* regions (Table 1), with *Diaporthellacorylina* (CBS 121124) as outgroup. The second analysis focused on the *Diaportheeres* complex based on *cal*, *tef1* and *tub2* loci (Table 3) according to recent publications (Gao et al. 2014, 2015, 2016, Udayanga et al. 2014b, Tanney et al. 2016, Fan et al. 2018), with *Diaporthecitri* (AR3405) as outgroup. Maximum Parsimony analysis was performed by a heuristic search option of 1000 random-addition sequences with a tree bisection and reconnection (TBR) algorithm. Maxtrees were set to 5000, branches of zero length were collapsed and all equally parsimonious trees were saved. Other calculated parsimony scores were tree length (TL), consistency index (CI), retention index (RI) and rescaled consistency (RC). Maximum Likelihood analysis was performed with a GTR site substitution model (Guindon et al. 2010). Branch support was evaluated with a bootstrapping (BS) method of 1000 replicates (Hillis and Bull 1993).

**Table 3.** Isolates and GenBank accession numbers used in the phylogenetic analyses of *Diaportheeres* complex.

Species	Isolate/culture collection	Host	Location	GenBank accession numbers		
				CAL	TEF1-α	TUB
* D. alleghaniensis *	CBS 495.72	* Betula alleghaniensis *	Canada	KC343249	GQ250298	KC843228
* D. alnea *	CBS 146.46	*Alnus* sp.	Netherlands	KC343250	KC343734	KC343976
	CBS 159.47	*Alnus* sp.	Netherlands	KC343251	KC343735	KC343977
	LCM22b.02a	*Alnus* sp.	USA	KJ435020	KJ210557	KJ420825
	LCM22b.02b	*Alnus* sp.	USA	KJ435021	KJ210558	KJ420826
*** D. betulina ***	**CFCC 52560**	*** Betula albo-sinensis ***	**China**	**MH121419**	**MH121537**	**MH121577**
	**CFCC 52561**	*** Betula costata ***	**China**	**MH121420**	**MH121538**	**MH121578**
	**CFCC 52562**	*** Betula platyphylla ***	**China**	**MH121421**	**MH121539**	**MH121579**
* D. bicincta *	CBS 121004	*Juglans* sp.	USA	KC343376	KC343860	KC344102
* D. biguttusis *	CGMCC 3.17081	* Lithocarpus glabra *	China	N/A^a^	KF576257	KF576306
* D. camptothecicola *	CFCC 51632	* Camptotheca acuminata *	China	KY228881	KY228887	KY228893
* D. celastrina *	CBS 139.27	*Celastrus* sp.	USA	KC343289	KC343773	KC344015
*** D. chensiensis ***	**CFCC 52567**	*** Abies chensiensis ***	**China**	**MH121426**	**MH121544**	**MH121584**
	**CFCC 52568**	*** Abies chensiensis ***	**China**	**MH121427**	**MH121545**	**MH121585**
* D. citri *	AR3405	*Citrus* sp.	USA	KC843157	KC843071	KC843187
* D. citrichinensis *	ZJUD034	*Citrus* sp.	China	KC843234	KC843071	KC843187
	ZJUD034B	*Citrus* sp.	China	KJ435042	KJ210562	KJ420829
* D. ellipicola *	CGMCC 3.17084	* Lithocarpus glabra *	China	N/A^a^	KF576245	KF576291
*** D. eres ***	AR5193	* Ulmus laevis *	Germany	KJ434999	KJ210550	KJ420799
	AR5196	* Ulmus laevis *	Germany	KJ435006	KJ210554	KJ420817
	DP0438	* Ulmus minor *	Austria	KJ435016	KJ210553	KJ420816
	LCM114.01a	*Ulmus* sp.	USA	KJ435027	KJ210545	KJ420787
	LCM114.01b	*Ulmus* sp.	USA	KJ435026	KJ210544	KJ420786
	FAU483	*Malus* sp.	Netherlands	KJ435022	JQ807422	KJ420827
	DAN001A	* Daphne laureola *	France	KJ434994	KJ210540	KJ420781
	DAN001B	* Daphne laureola *	France	KJ434995	KJ210541	KJ420782
	AR5197	*Rhododendron* sp.	Germany	KJ435014	KJ210552	KJ420812
	CBS 439.82	*Cotoneaster* sp.	UK	JX197429	GQ250341	JX275437
	AR3519	* Corylus avellana *	Austria	KJ435008	KJ210547	KJ420789
	FAU506	* Cornus florida *	USA	KJ435012	JQ807403	KJ420792
	FAU570	* Oxydendrum arboreum *	USA	KJ435025	JQ807410	KJ420794
	AR3723	* Rubus fruticosus *	Austria	KJ435024	JQ807354	KJ420793
	FAU522	* Sassafras albida *	USA	KJ435010	JQ807406	KJ420791
	DP0666	* Juglans cinerea *	USA	KJ435007	KJ210546	KJ420788
	DP0667	* Juglans cinerea *	USA	KC843155	KC843121	KC843229
	AR3560	*Viburnum* sp.	Austria	KJ435011	JQ807351	KJ420795
	AR5224	* Hedera helix *	Germany	KJ435036	KJ210551	KJ420802
	AR5231	* Hedera helix *	Germany	KJ435038	KJ210555	KJ420818
	AR5223	* Acer nugundo *	Germany	KJ435000	KJ210549	KJ420830
	CBS 109767	*Acer* sp.	Austria	KC343317	KC343801	KC344043
	DLR12a	* Vitis vinifera *	France	KJ434996	KJ210542	KJ420783
	DLR12b	* Vitis vinifera *	France	KJ434997	KJ210543	KJ420784
	AR4347	* Vitis vinifera *	Korea	KJ435030	JQ807356	KJ420805
	AR4355	*Prunus* sp.	Korea	KJ435035	JQ807359	KJ420797
	AR4367	*Prunus* sp.	Korea	KJ435019	JQ807364	KJ420824
	AR4346	* Prunus mume *	Korea	KJ435003	JQ807355	KJ420823
	AR4348	* Prunus persici *	Korea	KJ435004	JQ807357	JQ807357
	AR3669	* Pyrus pyrifolia *	Japan	KJ435002	JQ807415	KJ420808
*** D. eres ***	AR3670	* Pyrus pyrifolia *	Japan	KJ435001	JQ807416	KJ420807
	AR3671	* Pyrus pyrifolia *	Japan	KJ435017	JQ807417	KJ420814
	AR3672	* Pyrus pyrifolia *	Japan	KJ435023	JQ807418	KJ420819
	DP0591	* Pyrus pyrifolia *	New Zealand	KJ435018	JQ807395	KJ420821
	AR4369	* Pyrus pyrifolia *	Korea	KJ435005	JQ807366	KJ420813
	DP0180	* Pyrus pyrifolia *	New Zealand	KJ435029	JQ807384	KJ420804
	DP0179	* Pyrus pyrifolia *	New Zealand	KJ435028	JQ807383	KJ420803
	DP0590	* Pyrus pyrifolia *	New Zealand	KJ435037	JQ807394	KJ420810
	AR4373	* Ziziphus jujuba *	Korea	KJ435013	JQ807368	KJ420798
	AR4374	* Ziziphus jujuba *	Korea	KJ434998	JQ807369	KJ420785
	AR4357	* Ziziphus jujuba *	Korea	KJ435031	JQ807360	KJ420806
	AR4371	* Malus pumila *	Korea	KJ435034	JQ807367	KJ420796
	FAU532	* Chamaecyparis thyoides *	USA	KJ435015	JQ807408	KJ435015
	CBS 113470	* Castanea sativa *	Australia	KC343388	KC343872	KC344114
	AR4349	* Vitis vinifera *	Korea	KJ435032	JQ807358	KJ420822
	AR4363	*Malus* sp.	Korea	KJ435033	JQ807362	KJ420809
	**CFCC 52575**	*** Castanea mollissima ***	**China**	**N/A^a^**	**MH121552**	**MH121592**
	**CFCC 52576**	*** Castanea mollissima ***	**China**	**MH121432**	**MH121553**	**MH121593**
	**CFCC 52577**	*** Acanthopanax senticosus ***	**China**	**MH121433**	**MH121554**	**MH121594**
	**CFCC 52578**	***Sorbus* sp.**	**China**	**MH121434**	**MH121555**	**MH121595**
	**CFCC 52579**	*** Juglans regia ***	**China**	**N/A^a^**	**MH121556**	**N/A^a^**
	**CFCC 52580**	*** Melia azedarace ***	**China**	**N/A^a^**	**MH121557**	**MH121596**
	**CFCC 52581**	*** Rhododendron simsii ***	**China**	**N/A^a^**	**MH121558**	**MH121597**
* D. helicis *	AR5211	* Hedera helix *	France	KJ435043	KJ210559	KJ420828
* D. longicicola *	CGMCC 3.17089	* Lithocarpus glabra *	China	N/A^a^	KF576242	KF576291
* D. mahothocarpus *	CGMCC 3.15181	* Lithocarpus glabra *	China	N/A^a^	KC153087	KF576312
* D. maritima *	DAOMC 250563	* Picea rubens *	Canada	N/A^a^	N/A^a^	KU574616
* D. momicola *	MFLUCC 16-0113	* Prunus persica *	China	N/A^a^	KU557631	KU55758
* D. neilliae *	CBS 144. 27	*Spiraea* sp.	USA	KC343386	KC343870	KC344112
*** D. padina ***	**CFCC 52590**	*** Padus racemosa ***	**China**	**MH121443**	**MH121567**	**MH121604**
	**CFCC 52591**	*** Padus racemosa ***	**China**	**MH121444**	**MH121568**	**MH121605**
* D. phragmitis *	CBS 138897	* Phragmites australis *	China	N/A^a^	N/A^a^	KP004507
* D. pulla *	CBS 338.89	* Hedera helix *	Yugoslavia	KC343394	KC343878	KC344120
* D. vaccinii *	DF5032	* Vaccinium corymbosum *	USA	KC849457	JQ807380	KC843225
	FAU633	* Vaccinium macrocarpon *	USA	KC849456	JQ807413	KC843226
	FAU446	* Vaccinium macrocarpon *	USA	KC849455	JQ807398	KC843224
	CBS 160.32	* Vaccinium macrocarpon *	USA	KC343470	GQ250326	JX270436
	FAU 468	* Vaccinium macrocarpon *	USA	KC849458	JQ807399	KC843227

Newly sequenced material is indicated in bold type.

Bayesian inference (BI) analysis, employing a Markov chain Monte Carlo (MCMC) algorithm, was performed (Rannala and Yang 1996). MrModeltest v. 2.3 was used to estimate the best-fit model of nucleotide substitution model settings for each gene (Posada and Crandall 1998). Two MCMC chains started from random trees for 1,000,000 generations and trees were sampled every 100^th^ generation, resulting in a total of 10,000 trees. The first 25% of trees were discarded as the burn-in phase of each analysis. Branches with significant Bayesian posterior probabilities (BPP) were estimated in the remaining 7500 trees.

Sequences data were deposited in GenBank (Table 1). The multilocus sequence alignments were deposited in TreeBASE (www.treebase.org) as accession S22702 and S22703. The taxonomic novelties were deposited in MycoBank (Crous et al. 2004b).

## Results

### Collection of *Diaporthe* strains

Forty-two representative *Diaporthe* strains were isolated from 16 different host genera (Table 1) collected from six Provinces (Beijing, Heilongjiang, Jiangsu, Jiangxi, Shaanxi and Zhejiang) in China. All of these strains were isolated from symptomatic or non-symptomatic branches or twigs and preserved in the China Forestry Culture Collection Centre (CFCC).

### Phylogenetic analyses

**Figure 1.**
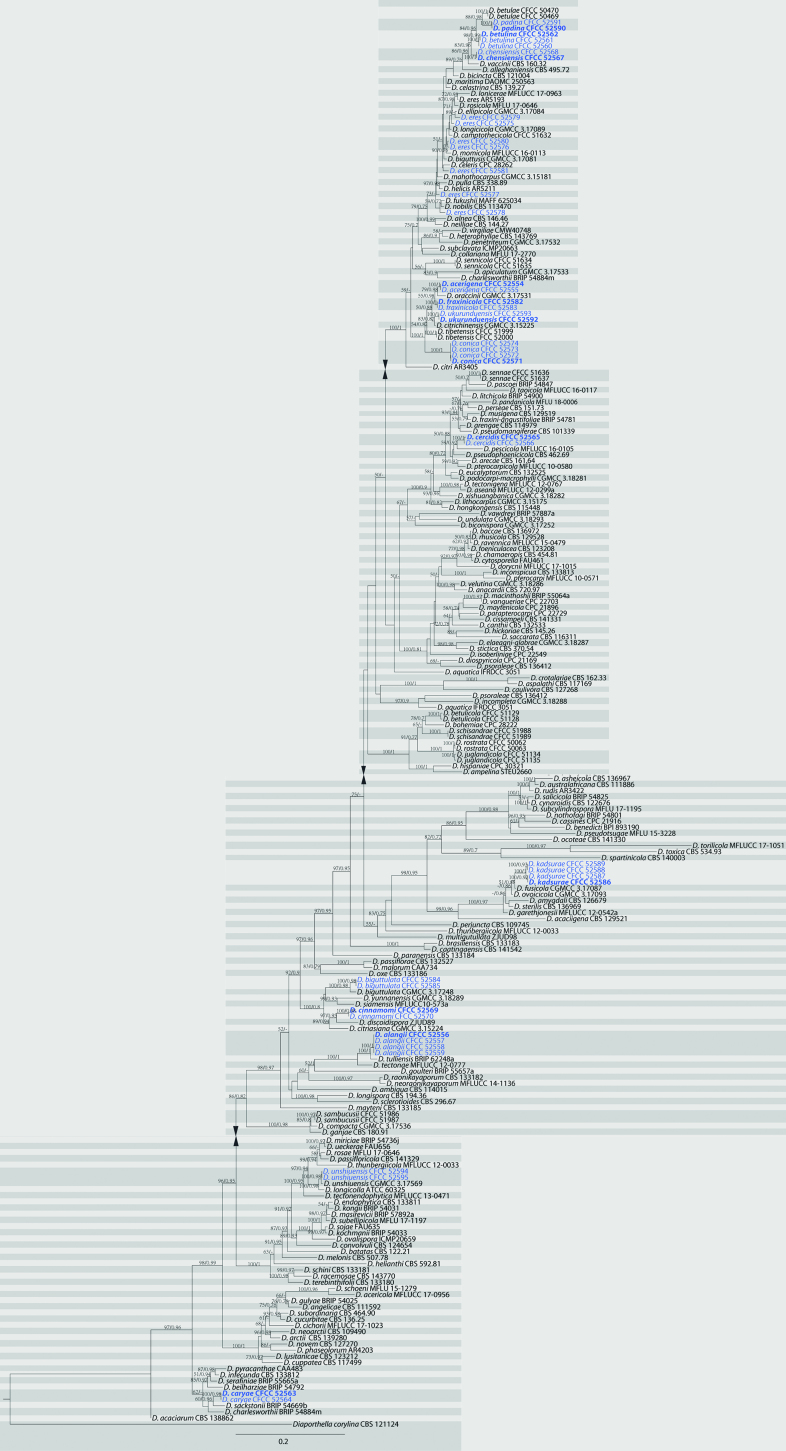
Phylogram of *Diaporthe* from a maximum likelihood analysis based on combined ITS, *cal*, *his3*, *tef1* and *tub2*. Values above the branches indicate maximum likelihood bootstrap (left, ML BP ≥ 50%) and bayesian probabilities (right, BI PP ≥ 0.70). The tree is rooted with *Diaporthellacorylina*. Strains in the current study are in blue.

The first sequences dataset for the ITS, *cal*, *his3*, *tef1*, and *tub2* was analysed in combination to infer the interspecific relationships within *Diaporthe*. The combined species phylogeny of the *Diaporthe* isolates consisted of 236 sequences, including the outgroup sequences of *Diaporthellacorylina* (culture CBS 121124). A total of 2948 characters including gaps (516 for ITS, 568 for *cal*, 520 for *his3*, 486 for *tef1* and 858 for *tub2*) were included in the phylogenetic analysis. The maximum likelihood tree, conducted by the GTR model, confirmed the tree topology and posterior probabilities of the Bayesian consensus tree. For the Bayesian analyses, MrModeltest suggested that all partitions should be analysed with dirichlet state frequency distributions. The following models were recommended by MrModeltest and used: GTR+I+G for ITS, *cal* and *his3*, HKY+I+G for *tef1* and *tub2*. The topology and branching order of ML were similar to BI analyses (Fig. 1). Based on the multi-locus phylogeny and morphology, 42 strains were assigned to 15 species, including 12 taxa which we describe here as new (Fig. 1).

**Figure 2.**
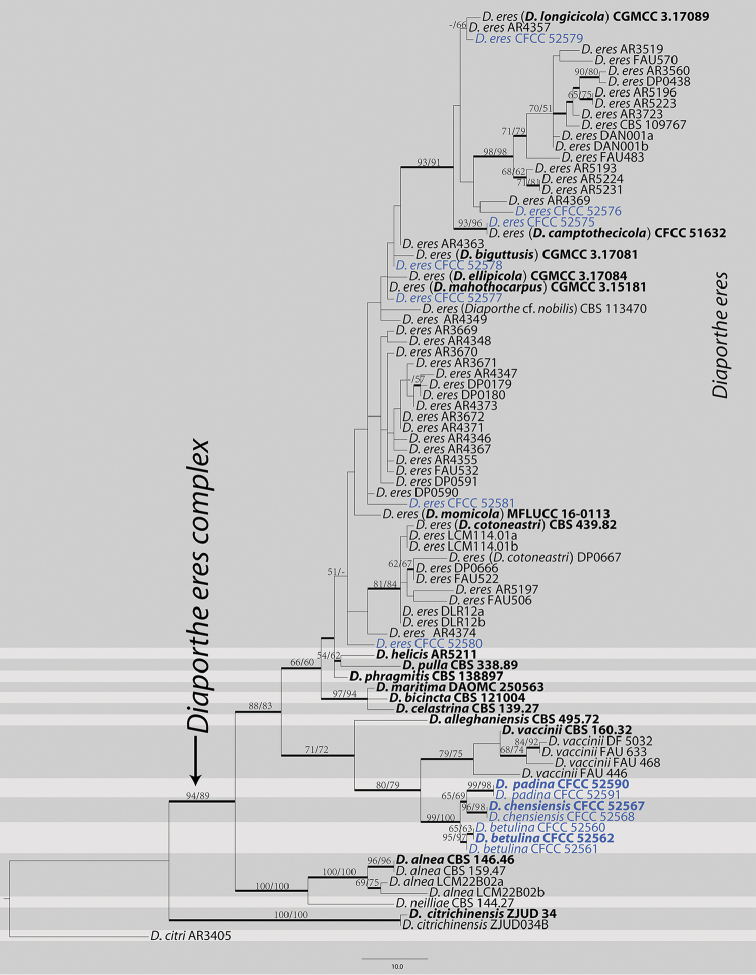
Phylogram of *Diaportheeres* complex based on combined *cal*, *tef1* and *tub2*. Values above the branches indicate maximum parsimony bootstrap (left, MP BP ≥ 50%) and maximum likelihood bootstrap (right, ML BP ≥ 50%). Values below branches represent posterior probabilities (BI PP ≥ 0.70) from Bayesian inference. The tree is rooted with *Diaporthecitri*. Strains in the current study are in blue. The ex-type/ex-epitype culture is in bold.

The second dataset with *cal*, *tef1* and *tub2* sequences were analysed to focus on the *Diaportheeres* complex. The alignment included 86 taxa, including the outgroup sequences of *Diaporthecitri* (Table 3). The aligned three-locus datasets included 1148 characters. Of these, 881 characters were constant, 105 variable characters were parsimony-uninformative and 162 characters were parsimony informative. The heuristic search using maximum parsimony (MP) generated 105 parsimonious trees (TL = 438, CI = 0.669, RI = 0.883, RC = 0.591), from which one was selected (Fig. 2). Based on the multi-locus phylogeny and morphology, seven strains were identified as *D.eres*, seven strains formed three distinct clades embedded in the *D.eres* complex, i.e. *D.betulina*, *D.chensiensis* and *D.padina*. MP and ML bootstrap support values above 50% are shown as first and second position, respectively. The branches with significant Bayesian posterior probability (≥ 0.70) in Bayesian analyses were thickened in the phylogenetic tree. The current results, based on the three genes (*cal*, *tef1* and *tub2*), suggest that *D.eres* clade could be separated from other species in this complex (Fig. 2). However, *D.biguttusis* (CGMCC 3.17081), *D.camptothecicola* (CFCC 51632), *D.ellipicola* (CGMCC 3.17084), *D.longicicola* (CGMCC 3.17089), *D.mahothocarpus* (CGMCC 3.15181) and *D.momicola* (MFLUCC 16-0113) were clustered in *D.eres* clade and thus treated as the synonyms of *D.eres* in the current study.

### Taxonomy

#### Diaporthe acerigena

Taxon classificationFungiDiaporthalesDiaporthaceae

C.M. Tian & Q. Yang sp. nov.

MB824703

Figure 3

##### Diagnosis.

*Diaportheacerigena* can be distinguished from the phylogenetically closely related species *D.oraccinii* in larger alpha conidia.

##### Holotype.

CHINA. Shaanxi Province: Qinling Mountain, on symptomatic twigs of *Acertataricum*, 27 June 2017, N. Jiang (holotype: BJFC-S1466; ex-type culture: CFCC 52554).

##### Etymology.

Named after the host genus on which it was collected, *Acer*.

##### Description.

On PDA: Conidiomata pycnidial, globose, solitary or aggregated, deeply embedded in the medium, erumpent, dark brown to black, 185–270 μm diam, whitish translucent to cream conidial drops exuding from the ostioles. Conidiophores 14.5–17 × 1.4–2.9 μm, cylindrical, hyaline, phiailidic, branched, straight to sinuous. Alpha conidia 7–10 × 2.1–2.9 μm (av. = 8.6 × 2.5 μm, n = 30), aseptate, hyaline, ellipsoidal, rounded at one end, slightly apex at the other end, usually with two-guttulate. Beta conidia not observed.

**Figure 3.**
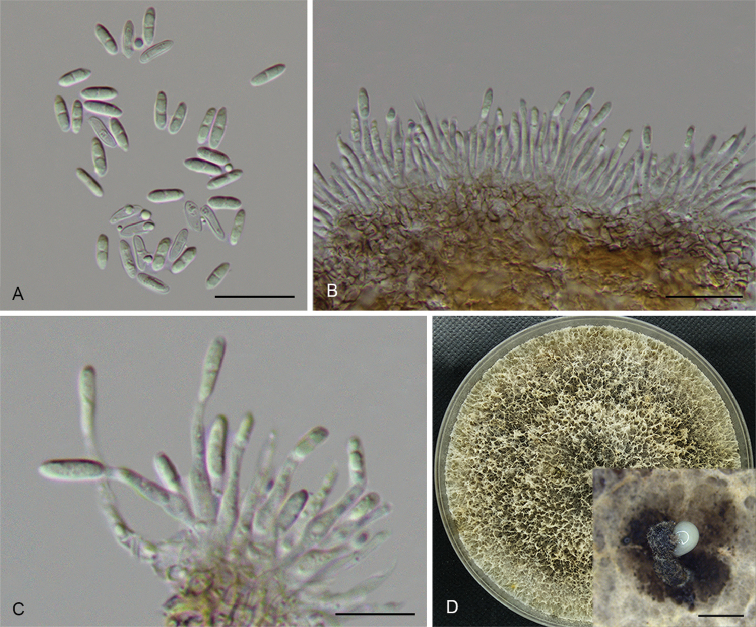
*Diaportheacerigena* (CFCC 52554) **A** Alpha conidia **B–C** Conidiophores **D** Culture on PDA and conidiomata. Scale bars: 20 μm (**A–C**), 200 μm (**D**).

##### Culture characters.

Cultures incubated on PDA at 25 °C in darkness. Colony at first white, becoming dark brown in the centre with age. Aerial mycelium white, dense, fluffy, with cream conidial drops exuding from the ostioles.

##### Additional specimens examined.

CHINA. Shaanxi Province: Qinling Mountain, on symptomatic twigs of *Acertataricum*, 27 June 2017, N. Jiang, living culture CFCC 52555 (BJFC-S1467).

##### Notes.

Two strains representing *D.acerigena* cluster in a well-supported clade and appear most closely related to *D.oraccinii*. *Diaportheacerigena* can be distinguished from *D.oraccinii* based on ITS, *his3*, *tef1* and *tub2* loci (5/469 in ITS, 8/429 in *his3*, 8/326 in *tef1* and 5/358 in *tub2*). Morphologically, *D.acerigena* differs from *D.oraccinii* in the longer and larger alpha conidia (8.6 × 2.5 vs. 6.6 × 1.9 μm) (Gao et al. 2016).

#### Diaporthe alangii

Taxon classificationFungiDiaporthalesDiaporthaceae

C.M. Tian & Q. Yang sp. nov.

MB824704

Figure 4

##### Diagnosis.

*Diaporthealangii* can be distinguished from the phylogenetically closely related species *D.tectonae* and *D.tulliensis* by the size of conidiophores and alpha conidia.

##### Holotype.

CHINA. Zhejiang Province: Tianmu Mountain, on symptomatic branches of *Alangiumkurzii*, 19 Apr. 2017, Q. Yang (holotype: BJFC-S1468; ex-type culture: CFCC 52556).

##### Etymology.

Named after the host genus on which it was collected, *Alangium*.

##### Description.

Conidiomata pycnidial, immersed in bark, scattered, erumpent through the bark surface, discoid, with a solitary undivided locule. Ectostromatic disc black, one ostiole per disc, 135–330 μm diam. Locule circular, undivided, 290–445 μm diam. Conidiophores 6–12 × 1.4–2 μm, cylindrical, hyaline, phiailidic, unbranched, straight. Alpha conidia 6.5–8 × 2 μm (av. = 7 × 2 μm, n = 30), aseptate, hyaline, ellipsoidal, biguttulate, mostly with one end obtuse and the other acute, occasionally submedian constriction. Beta conidia not observed.

**Figure 4.**
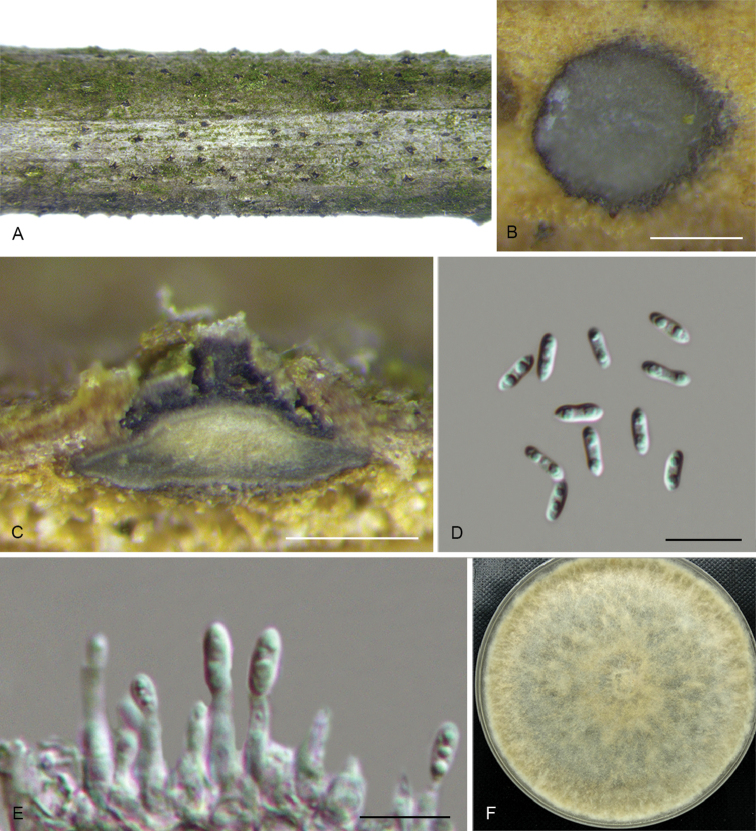
*Diaporthealangii* (CFCC 52556) **A** Habit of conidiomata on branches **B** Transverse section of conidioma **C** Longitudinal section of conidioma **D** Alpha conidia **E** Conidiophores **F** Culture on PDA. Scale bars: 200 μm (**B–C**), 10 μm (**D–E**).

##### Culture characters.

Cultures incubated on PDA at 25 °C in darkness. Colony initially white, producing beige pigment after 7–10 d. The colony is flat, felty with a thick texture at the centre and marginal area, with thin texture in the middle, lacking aerial mycelium, conidiomata absent.

##### Additional specimens examined.

CHINA. Zhejiang Province: Tianmu Mountain, on symptomatic branches of *Alangiumkurzii*, 19 Apr. 2017, Q. Yang, living culture CFCC 52557 (BJFC-S1469); ibid. living culture CFCC 52558 (BJFC-S1470); ibid. living culture CFCC 52559 (BJFC-S1471).

##### Notes.

Four isolates clustered in a clade distinct from its closest phylogenetic neighbour, *D.tectonae* and *D.tulliensis. Diaporthealangii* can be distinguished from *D.tectonae* in *cal*, *tef1* and *tub2* loci (6/458 in *cal*, 4/308 in *tef1* and 11/407 in *tub2*); from *D.tulliensis* in ITS, tef1 and tub2 loci (6/462 in ITS, 8/308 in *tef1* and 10/701 in *tub2*). Morphologically, *D.alangii* differs from *D.tectonae* in shorter conidiophores (6–12 vs. 11–18 μm) and longer alpha conidia (6.5–8 vs. 5.5–6 μm); from *D.tulliensis* in shorter conidiophores (6–12 vs. 15–20 μm) (Crous et al. 2015, Doilom et al. 2017).

#### Diaporthe betulina

Taxon classificationFungiDiaporthalesDiaporthaceae

C.M. Tian & Q. Yang sp. nov.

MB824705

Figure 5

##### Diagnosis.

*Diaporthebetulina* can be distinguished from the phylogenetically closely related species *D.betulae* in smaller locule and wider alpha conidia.

##### Holotype.

CHINA. Heilongjiang Province: Yichun city, on symptomatic branches of *Betulaplatyphylla*, 27 July 2016, Q. Yang (holotype: BJFC-S1472; ex-type culture: CFCC 52562).

##### Etymology.

Named after the host genus on which it was collected, *Betula*.

##### Description.

Conidiomata pycnidial, conical, immersed in bark, scattered, erumpent through the bark surface, with a solitary undivided locule. Ectostromatic disc brown to black, one ostiole per disc, 290–645 μm diam. Ostiole medium black, up to the level of disc. Locule undivided, 670–905 μm diam. Conidiophores 12.5–17.5 × 1.5–2 μm, cylindrical, hyaline, phiailidic, branched, straight or slightly curved. Alpha conidia hyaline, aseptate, ellipsoidal to fusiform, 0–2-guttulate, sometimes acute at both ends, 8–10 × 2.5–3 μm (av. = 9 × 2.6 μm, n = 30). Beta conidia hyaline, aseptate, filiform, straight or hamate, eguttulate, base subtruncate, tapering towards one apex, 26–32.5 × 1 µm (av. = 30 × 1 µm, n = 30).

**Figure 5.**
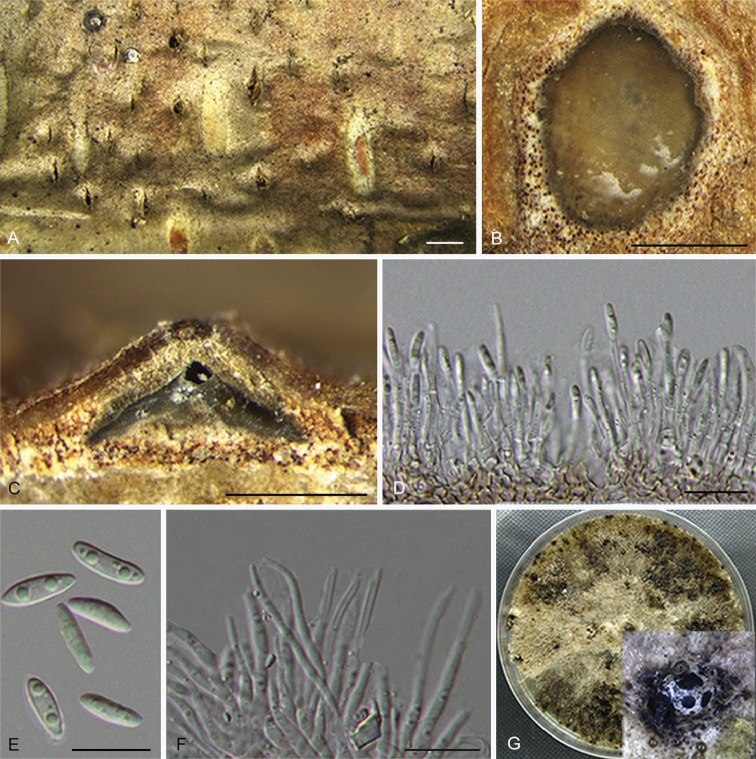
*Diaporthebetulina* (CFCC 52562) **A** Habit of conidiomata on branches **B** Transverse section of conidioma **C** Longitudinal section of conidioma **D** Conidiophores **E** Alpha conidia **F** Beta conidia **G** Culture on PDA and conidiomata. Scale bars: 500 μm (**A–C**), 10 μm (**D–F**).

##### Culture characters.

Cultures incubated on PDA at 25 °C in darkness. Colony flat with white felty aerial mycelium, turning white to dark brown aerial mycelium, conidiomata irregularly distributed on the agar surface.

##### Additional specimens examined.

CHINA. Heilongjiang Province: Yichun city, on symptomatic branches of *Betulaalbo-sinensis*, 27 July 2016, Q. Yang, living culture CFCC 52560 (BJFC-S1473); on symptomatic branches of *Betulacostata*, 27 July 2016, Q. Yang, living culture CFCC 52561 (BJFC-S1474).

##### Notes.

*Diaporthebetulina* was isolated from *Betula* spp. cankers in Heilongjiang Province. Three strains representing *D.betulina* cluster in a well-supported clade and appear most closely related to *D.betulae*, which was also isolated from *Betulaplatyphylla* in Sichuang Province (Du et al. 2016). *Diaporthebetulina* can be distinguished based o*n* ITS, *his3*, *tef1* and *tub2* loci from *D.betulae* (11/461 in ITS, 9/453 in *his3*, 12/336 in *tef1* and 7/695 in *tub2*). Morphologically, *D.betulina* differs from *D.betulae* in smaller locule (470–945 vs. 600–1250 μm) and wider alpha conidia (3–4 vs. 2.5–3 μm) (Du et al. 2016).

#### Diaporthe biguttulata

Taxon classificationFungiDiaporthalesDiaporthaceae

F. Huang, K.D. Hyde & H.Y. Li, 2015

Figure 6

##### Description.

Conidiomata pycnidial, immersed in bark, scattered, erumpent through the bark surface, discoid, with a single locule. Ectostromatic disc dark brown, one ostiole per disc, 160–320 μm diam. Locule undivided, 235–350 μm diam. Conidiophores 8.5–11 × 1.5 μm, cylindrical, hyaline, branched, straight or slightly curved, tapering towards the apex. Alpha conidia hyaline, aseptate, ellipsoidal to oval, 2-guttulate, usually rounded at both ends, occasionally with one end acute, 7–8.5 × 1.5–2 μm (av. = 6.5 × 2.6 μm, n = 30). Beta conidia not observed.

**Figure 6.**
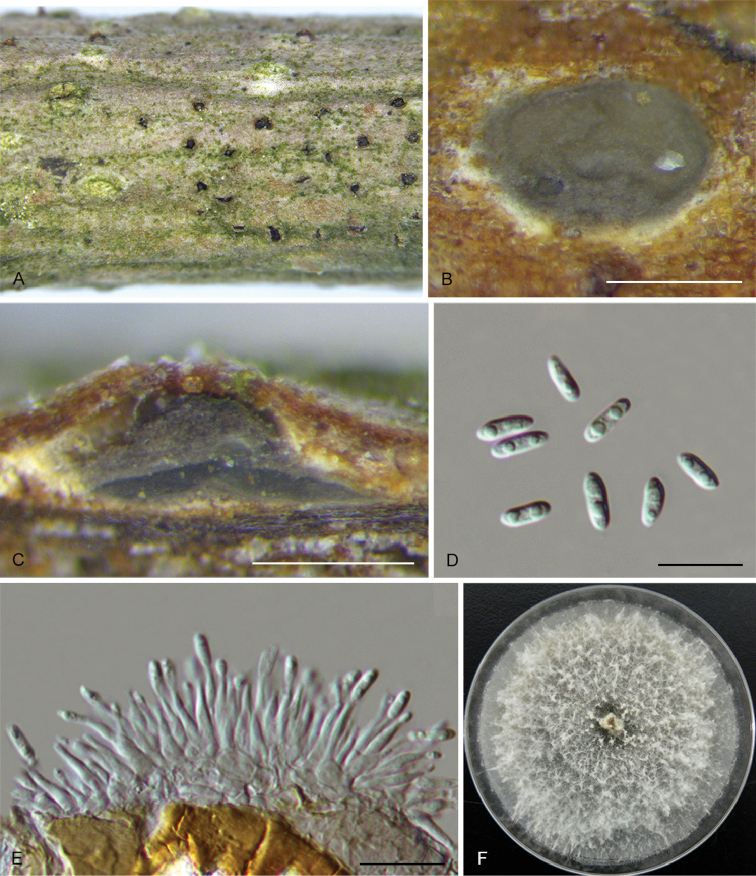
*Diaporthebiguttulata* (CFCC 52584) **A** Habit of conidiomata on branches **B** Transverse section of conidioma **C** Longitudinal section of conidioma **D** Alpha conidia **E** Conidiophores **F** Culture on PDA. Scale bars: 200 μm (**B–C**), 10 μm (**D–E**).

##### Culture characters.

Cultures incubated on PDA at 25 °C in darkness. Colony originally flat with white aerial mycelium, becoming pale grey, with dense aerial mycelium in the centre and sparse aerial mycelium at the marginal area, conidiomata absent.

##### Specimens examined.

CHINA. Zhejiang Province: Tianmu Mountain, on symptomatic branches of *Juglansregia*, 20 Apr. 2017, Q. Yang, living culture CFCC 52584 and CFCC 52585 (BJFC-S1504).

##### Notes.

*Diaporthebiguttulata* was originally described from a healthy branch of *Citruslimon* in Yunnan Province, China (Huang et al. 2015). In the present study, two isolates (CFCC 52584 and CFCC 52585) from symptomatic branches of *Juglansregia* were congruent with *D.biguttulata* based on morphology and DNA sequences data (Fig. 1). We therefore describe *D.biguttulata* as a known species for this clade.

#### Diaporthe caryae

Taxon classificationFungiDiaporthalesDiaporthaceae

C.M. Tian & Q. Yang sp. nov.

MB824706

Figure 7

##### Diagnosis.

*Diaporthecaryae* differs from its closest phylogenetic neighbour, *D.charlesworthii* and *D.sackstonii*, in ITS, *tef1* and *tub2* loci based on the alignments deposited in TreeBASE.

##### Holotype.

CHINA. Jiangsu Province: Nanjing city, on symptomatic twigs of *Caryaillinoensis*, 10 Nov. 2015, Q. Yang (holotype: BJFC-S1476; ex-type culture: CFCC 52563).

##### Etymology.

Named after the host genus on which it was collected, *Carya*.

##### Description.

Conidiomata pycnidial, immersed in bark, scattered, slightly erumpent through the bark surface, nearly flat, discoid, with a solitary undivided locule. Ectostromatic disc brown to black, one ostiole per disc. Locule undivided, 310–325 μm diam. Conidiophores 7–11 × 1.4–2.2 μm, cylindrical, phialidic, unbranched, sometimes inflated. Alpha conidia hyaline, aseptate, ellipsoidal or fusiform, eguttulate, obtuse at both ends, 7–8.5 × 2.1–2.5 μm (av. = 8 × 2.3 μm, n = 30). Beta conidia hyaline, aseptate, filiform, straight or hamate, eguttulate, base subtruncate, tapering towards one apex, 15.5–34 × 1.1–1.4 µm (av. = 27.5 × 1.2 µm, n = 30).

**Figure 7.**
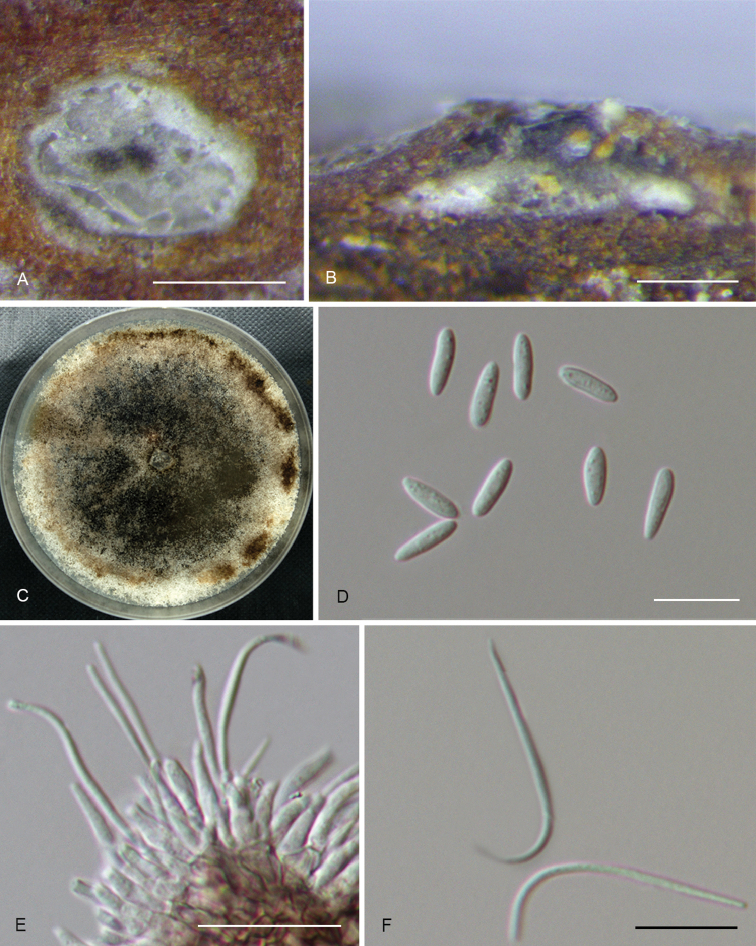
*Diaporthecaryae* (CFCC 52563) **A** Transverse section of conidioma **B** Longitudinal section of conidioma **C** Culture on PDA**D** Alpha conidia **E** Conidiophores **F** Beta conidia. Scale bars: 200 μm (**A**), 100 μm (**B**), 10 μm (**D, F**), 20 μm (**E**).

##### Culture characters.

Cultures incubated on PDA at 25 °C in darkness. Colony at first flat with white felty mycelium, becoming black in the centre and black at the marginal area with age, conidiomata not observed.

##### Additional specimens examined.

CHINA. Jiangsu Province: Nanjing city, on symptomatic twigs of *Caryaillinoensis*, 10 Nov. 2015, Q. Yang, living culture CFCC 52564 (BJFC-S1477).

##### Notes.

Two strains representing *D.caryae* cluster in a well-supported clade and appear closely related to *D.charlesworthii* and *D.sackstonii. Diaporthecaryae* can be distinguished based o*n* ITS, *tef1* and *tub2* loci from *D.charlesworthii* (50/468 in ITS, 107/338 in *tef1* and 90/707 in *tub2*); from *D.sackstonii* (4/440 in ITS, 13/340 in *tef1* and 23/701 in *tub2*). Morphologically, *D.caryae* can be distinguished from *D.charlesworthii* by its shorter conidiophores (7–11 vs. 15–35 μm); from *D.sackstonii* by its longer alpha conidia (7–8.5 vs. 6–7 μm) (Thompson et al. 2015).

#### Diaporthe cercidis

Taxon classificationFungiDiaporthalesDiaporthaceae

C.M. Tian & Q. Yang sp. nov.

MB824707

Figure 8

##### Diagnosis.

*Diaporthecercidis* can be distinguished from the phylogenetically closely related species *D.pescicola* in larger alpha conidia.

##### Holotype.

CHINA. Jiangsu Province: Nanjing city, on twigs and branches of *Cercischinensis*, 11 Nov. 2015, Q. Yang (holotype: BJFC-S1478; ex-type culture: CFCC 52565).

##### Etymology.

Named after the host genus on which it was collected, *Cercis*.

##### Description.

Conidiomata pycnidial, immersed in bark, scattered, slightly erumpent through the bark surface, nearly flat, discoid, with a solitary undivided locule. Ectostromatic disc grey to brown, one ostiole per disc. Locule circular, undivided, 135–200 μm diam. Conidiophores 7–17 × 1.4–2.1 μm, phialidic, unbranched, straight or slightly curved, tapering towards the apex. Alpha conidia hyaline, aseptate, fusiform to oval, biguttulate, 6.5–10 × 3–3.5 μm (av. = 8.6 × 3.3 μm, n = 30). Beta conidia hyaline, aseptate, filiform, straight or hamate, eguttulate, 20–28.5 × 1–1.3 µm (av. = 25.5 × 1.2 µm, n = 30).

**Figure 8.**
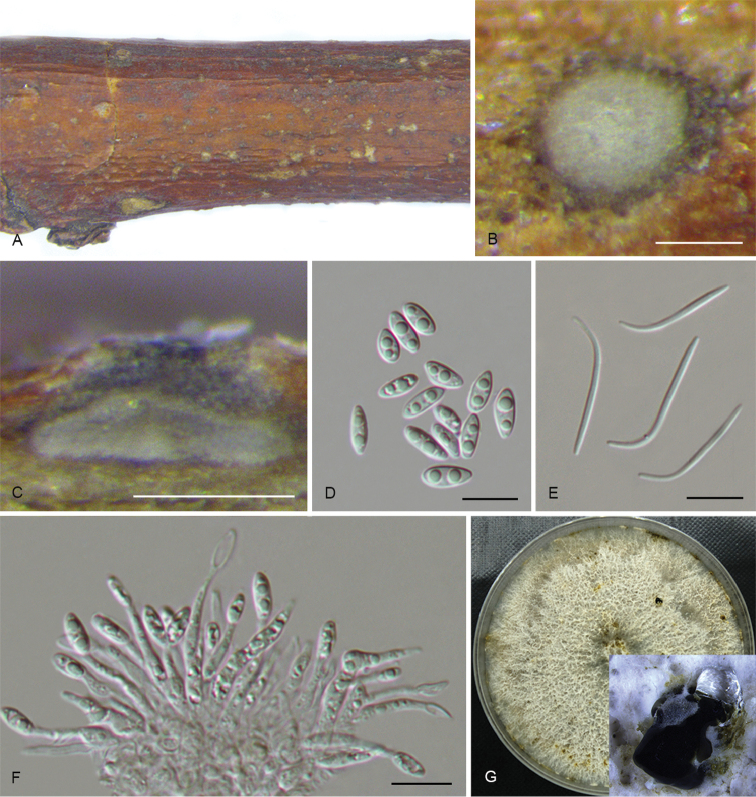
*Diaporthecercidis* (CFCC 52565) **A** Habit of conidiomata on branches **B** Transverse section of conidioma **C** Longitudinal section of conidioma **D** Alpha conidia **E** Beta conidia **F** Conidiophores **G** Culture on PDA and conidiomata. Scale bars: 100 μm (**B–C**), 10 μm (**D–F**).

##### Culture characters.

Cultures incubated on PDA at 25 °C in darkness showed colony at first white, becoming pale brown with yellowish dots with age, flat, with dense and felted mycelium, with visible solitary or aggregated conidiomata at maturity.

##### Additional specimens examined.

CHINA. Jiangsu Province: Yangzhou city, on twigs and branches of *Ginkgobiloba*, 11 Nov. 2015, N. Jiang, living culture CFCC 52566 (BJFC-S1479).

##### Notes.

*Diaporthecercidis* is distinguished from *D.pescicola* in the ITS, *cal* and *tef1* loci (13/458 in ITS, 47/442 in *cal* and 6/328 in *tef1*). Morphologically, *D.cercidis* differs from *D.pescicola* in shorter conidiophores (7–17 vs. 21–35 μm) and larger alpha conidia (6.5–10 × 3–3.5 vs. 6–8.5 × 2–3 μm) (Dissanayake et al. 2017a).

#### Diaporthe chensiensis

Taxon classificationFungiDiaporthalesDiaporthaceae

C.M. Tian & Q. Yang sp. nov.

MB824708

Figure 9

##### Diagnosis.

*Diaporthechensiensis* differs from its closest phylogenetic neighbour, *D.vaccinii*, in ITS, *cal*, *his3* and *tef1* loci based on the alignments deposited in TreeBASE.

##### Holotype.

CHINA. Shaanxi Province: Ningshan County, Huoditang forest farm, on symptomatic twigs of *Abieschensiensis*, 5 July 2017, Q. Yang (holotype: BJFC-S1480; ex-type culture: CFCC 52567).

##### Etymology.

Named after the host species on which it was collected, *chensiensis*.

##### Description.

Conidiomata pycnidial, immersed in bark, scattered, slightly erumpent through the bark surface, discoid, with a single locule. Ectostromatic disc white to brown, one ostiole per disc, 200–325 μm diam. Locule undivided, 385–540 μm diam. Conidiophores 8.5–13 × 2–3 μm, cylindrical, hyaline, phiailidic, unbranched, straight or slightly curved, tapering towards the apex. Alpha conidia hyaline, aseptate, smooth, ellipsoidal, biguttulate, rounded at both ends, 6.5–11 × 2–2.2 μm (av. = 8.5 × 2.1 μm, n = 30). Beta conidia present on the host, hyaline, eguttulate, smooth, filiform, hamate, 21–28.5 × 0.8–1.1 μm (av. = 25 × 1 μm, n = 30).

**Figure 9.**
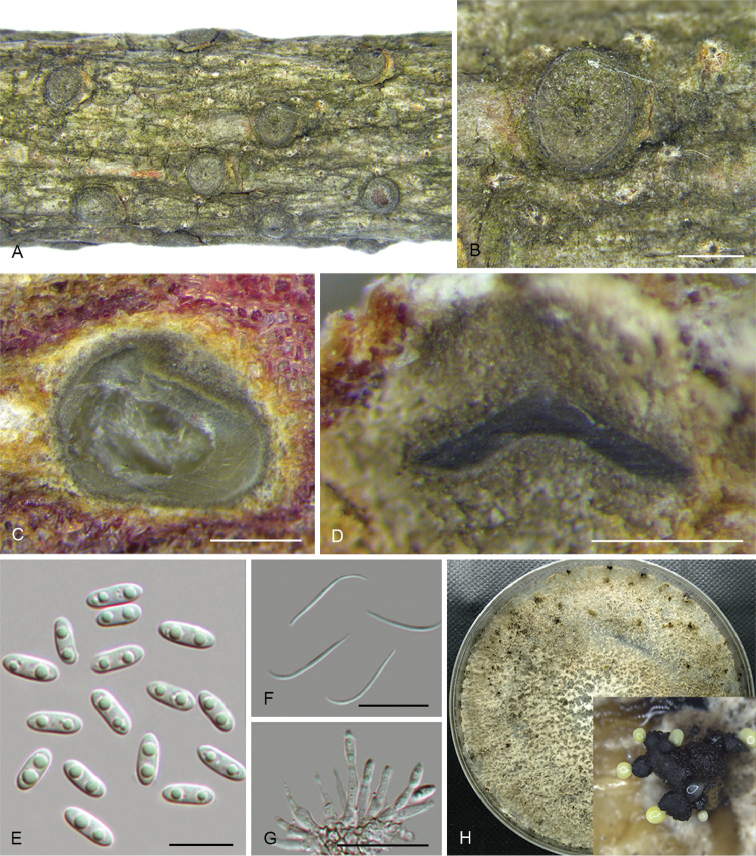
*Diaporthechensiensis* (CFCC 52567) **A–B** Habit of conidiomata on branches **C** Transverse section of conidioma **D** Longitudinal section of conidioma **E** Alpha conidia **F** Beta conidia **G** Conidiophores **H** Culture on PDA and conidiomata. Scale bars: 500 μm (**B**), 200 μm (**C–D**), 10 μm (**E**), 20 μm (**F**).

##### Culture characters.

Cultures incubated on PDA at 25 °C in darkness. Colony originally flat with white felted aerial mycelium, becoming light brown mycelium due to pigment formation, conidiomata irregularly distributed over agar surface, with yellowish conidial drops exuding from the ostioles.

##### Additional specimens examined.

CHINA. Shaanxi Province: Ningshan County, Huoditang forest farm, on symptomatic twigs of *Abieschensiensis*, 5 July 2017, Q. Yang, living culture CFCC 52568 (BJFC-S1481).

##### Notes.

*Diaporthechensiensis* occurs in an independent clade (Fig. 1) and is phylogenetically distinct from *D.vaccinii*. *Diaporhechensiensis* can be distinguished from *D.vaccinii* by 57 nucleotides in concatenated alignment, in which 14 were distinct in the ITS region, 13 in the *cal* region, 10 in the *his3* region, 15 in the *tef1* region and 15 in the *tub2* region. Although this species belongs to the *D.eres* complex, it is, however, distinct from the known species within the complex (Fig. 2).

#### Diaporthe cinnamomi

Taxon classificationFungiDiaporthalesDiaporthaceae

C.M. Tian & Q. Yang sp. nov.

MB824709

Figure 10

##### Diagnosis.

*Diaporthecinnamomi* differs from its closest phylogenetic species *D.discoidispora* in ITS, *his3* and *tef1* loci based on the alignments deposited in TreeBASE.

##### Holotype.

CHINA. Zhejiang Province: Linan city, on symptomatic twigs of *Cinnamomum* sp., 22 Apr. 2017, Q. Yang (holotype: BJFC-S1482; ex-type culture: CFCC 52569).

##### Etymology.

Named after the host genus on which it was collected, *Cinnamomum*.

##### Description.

On PDA: Conidiomata pycnidial, globose, solitary or aggregated, deeply embedded in the substrate, erumpent, dark brown to black, 170–235 μm diam., whitish translucent to cream conidial drops exuding from the ostioles. Conidiophores 11–25 × 1.5–2 μm, cylindrical, hyaline, branched, straight or curved, tapering towards the apex. Alpha conidia hyaline, aseptate, ellipsoidal to oval, biguttulate, rounded at both ends, 5–7 × 2.5–3 μm (av. = 6 × 2.9 μm, n = 30). Beta conidia not observed.

**Figure 10.**
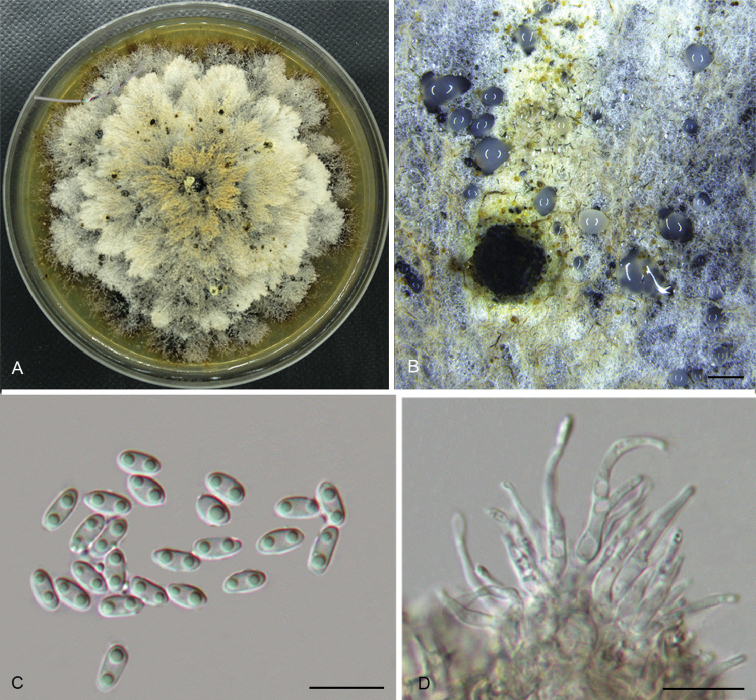
*Diaporthecinnamomi* (CFCC 52569) **A** Culture on PDA**B** Conidiomata **C** Alpha conidia **D** Conidiophores. Scale bars: 200 μm (**B**), 10 μm (**C–D**).

##### Culture characters.

Cultures incubated on PDA at 25 °C in darkness showed colony originally flat with white felty mycelium, developing petaloid mycelium after 7–10 d and turning yellowish at the centre and brownish at the marginal area after 15 d. Conidiomata erumpent at maturity.

##### Additional material examined.

CHINA. Zhejiang Province: Linan city, on symptomatic twigs of *Cinnamomum* sp., 22 Apr. 2017, Q. Yang, living culture CFCC 52570 (BJFC-S1483).

##### Notes.

*Diaporthecinnamomi* comprises strains CFCC 52569 and CFCC 52570 closely related to *D.discoidispora* in the combined phylogenetic tree (Fig. 1). *Diaporthecinnamomi* can be distinguished based on ITS, *his3* and *tef1* loci from *D.discoidispora* (4/460 in ITS, 17/448 in *his3* and 38/339 in *tef1*).

#### Diaporthe conica

Taxon classificationFungiDiaporthalesDiaporthaceae

C.M. Tian & Q. Yang sp. nov.

MB824710

Figure 11

##### Diagnosis.

*Diaportheconica* is phylogenetically and morphologically distinct from *D.rostrata*, in smaller locule and alpha conidia.

##### Holotype.

CHINA. Zhejiang Province: Tianmu Mountain, on symptomatic branches of *Alangiumchinense*, 20 Apr. 2017, Q. Yang (holotype: BJFC-S1484; ex-type culture: CFCC 52571).

##### Etymology.

Named after the conical conidiomata.

##### Description.

Conidiomata pycnidial, 420–580 μm diam., solitary and with single necks erumpent through the host bark. Tissue around the neck is conical. Locule oval, undivided, 385–435 μm diam. Conidiophores reduced to conidiogenous cells. Conidiogenous cells unbranched, straight or sinuous, apical or base sometimes swelling, 19–23.5 × 2.8 μm. Alpha conidia hyaline, aseptate, ellipsoidal, biguttulate, 5.5–7 × 2.3–3 μm (av. = 6.5 × 2.6 μm, n = 30). Beta conidia not observed.

**Figure 11.**
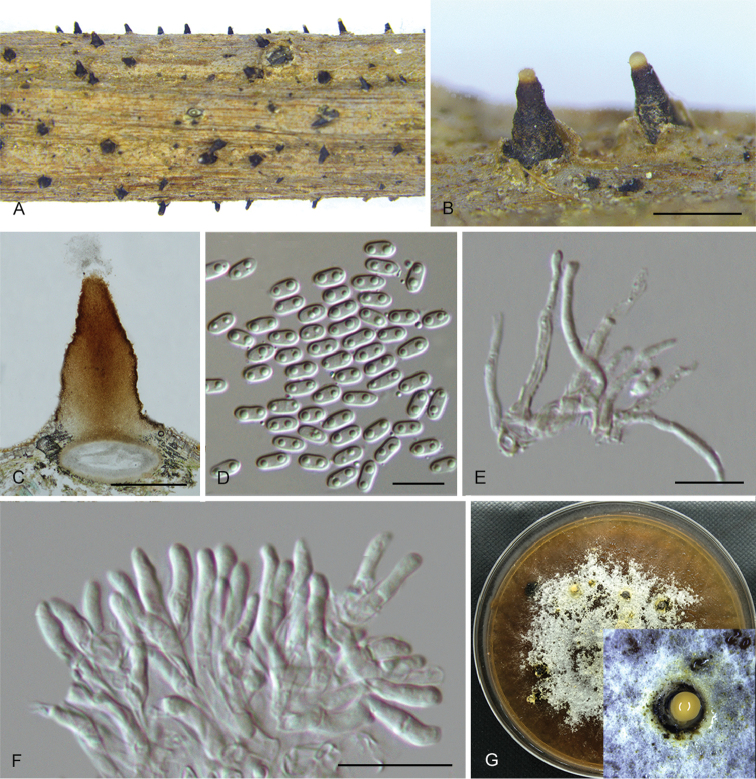
*Diaportheconica* (CFCC 52571) **A–B** Habit of conidiomata on branches **C** Longitudinal section of conidioma **D** Alpha conidia **E–F** Conidiophores **G** Culture on PDA and conidiomata. Scale bars: 300 μm (**B–C**), 10 μm (**D–F**).

##### Culture characters.

Cultures incubated on PDA at 25 °C in darkness. Colony white to yellowish, with dense and felted mycelium, lacking aerial mycelium, with maize-coloured conidial drops exuding from the ostioles.

##### Additional material examined.

CHINA. Zhejiang Province: Tianmu Mountain, on symptomatic branches of *Alangiumchinense*, 20 Apr. 2017, Q. Yang, living culture CFCC 52572 (BJFC-S1485); ibid. living culture CFCC 52573 (BJFC-S1486); ibid. living culture CFCC 52574 (BJFC-S1487).

##### Notes.

Four isolates clustered in a clade distinct from further *Diaporthe* species based on DNA sequence data. Morphologically, this species is characterised by conical conidiomata, which is similar with *D.rostrata* from *Juglansmandshurica*. However, *D.conica* differs from *D.rostrata* by having smaller locule and alpha conidia (310–385 vs. 620–1100 μm in locule; 5.5–7 × 2.3–3 vs. 8.5–11.5 × 4–5 μm in alpha conidia) (Fan et al. 2015).

#### Diaporthe eres

Taxon classificationFungiDiaporthalesDiaporthaceae

Nitschke, 1870

Figure 12

= Diaporthebiguttusis Y.H. Gao & L. Cai, 2015.
= Diaporthecamptothecicola C.M. Tian & Qin Yang, 2017.
= Diaportheellipicola Y.H. Gao & L. Cai, 2015.
= Diaporthelongicicola Y.H. Gao & L. Cai, 2015
= Diaporthemahothocarpus (Y.H. Gao, W. Sun & L. Cai) Y.H. Gao & L. Cai, 2015.
= Diaporthemomicola Dissan., J.Y. Yan, Xing H. Li & K.D. Hyde, 2017.

##### Description.

Conidiomata pycnidial, immersed in bark, erumpent through the bark surface, serried, with a single locule. Ectostromatic disc obviously, brown to black, with one ostiole per disc, 245–572 μm diam. Ostiole medium black, up to the level of disc. Locule circular, undivided, 335–450 μm diam. Conidiophores 10.5–19 × 1–1.5 μm, cylindrical, hyaline, unbranched, straight or slightly sinuous. Conidiogenous cells phialidic, cylindrical, terminal. Alpha conidia hyaline, aseptate, ellipsoidal to lanceolate, one guttulate at each end, 6–7.5 × 1.5–2.5 μm (av. = 6.5 × 2 μm, n = 30). Beta conidia not observed.

**Figure 12.**
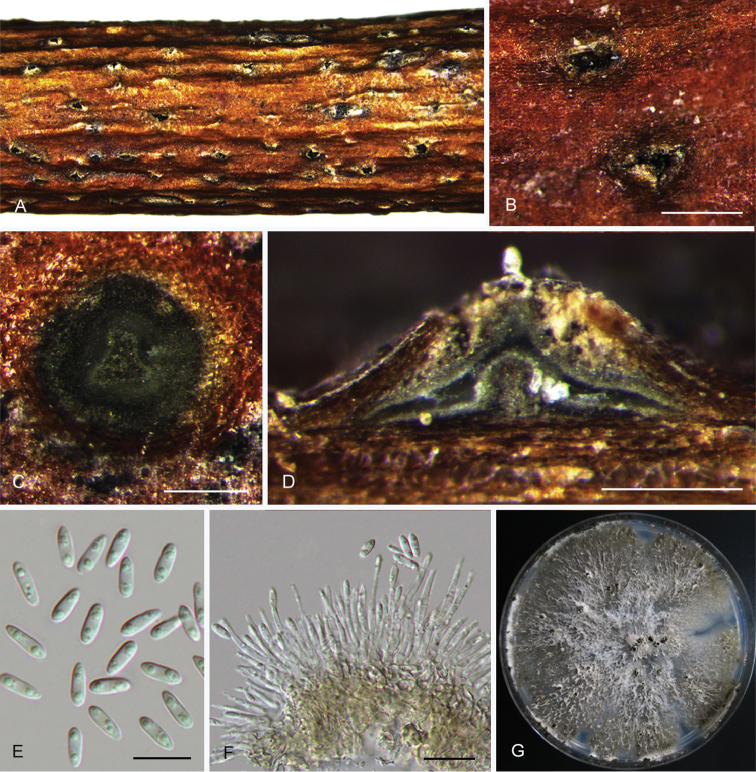
*Diaportheeres* (CFCC 52575) **A–B** Habit of conidiomata on branches **C** Transverse section of conidioma **D** Longitudinal section of conidioma **E** Alpha conidia **F** Conidiophores **G** Culture on PDA and conidiomata. Scale bars: 500 μm (**B**), 200 μm (**C–D**), 10 μm (**E–F**).

##### Culture characters.

Cultures on PDA incubated at 25 °C in darkness. Colony with white felty aerial mycelium, becoming white felted aerial mycelium in the centre and grey-brown mycelium at the marginal area, conidiomata irregularly distributed over agar surface.

##### Specimens examined.

CHINA. Beijing: Pinggu district, on symptomatic branches of *Castaneamollissima*, 1 Nov. 2016, N. Jiang, living culture CFCC 52576 (BJFC-S1489); ibid. living culture CFCC 52577 (BJFC-S1490). Heilongjiang Province: Liangshui Nature Reserve, on symptomatic twigs of *Acanthopanaxsenticosus*, 29 July 2016, Q. Yang, living culture CFCC 52580 (BJFC-S1493). Heilongjiang Province: Harbin city, Botanical garden, on symptomatic twigs of *Sorbus* sp., 2 Aug. 2016, Q. Yang, living culture CFCC 52575 (BJFC-S1488). Shaanxi Province: Zhashui County, on symptomatic branches of *Juglansregia*, 29 July 2016, Q. Yang, living culture CFCC 52579 (BJFC-S1492). Zhejiang Province: Yangzhou city, on symptomatic twigs of *Meliaazedarace*, 8 July 2017, N. Jiang, living culture CFCC 52578 (BJFC-S1491). Zhejiang Province: Tianmu Mountain, on symptomatic twigs of *Rhododendronsimsii*, 20 Apr. 2017, Q. Yang, living culture CFCC 52581 (BJFC-S1494).

##### Notes.

*Diaportheeres*, the type species of the genus, was described by Nitschke (1870) on *Ulmus* sp. collected in Germany, which has a widespread distribution and a broad host range as a pathogen, endophyte or saprobe causing leaf spots, stem cankers and diseases of woody plants (Udayanga et al. 2014b). Fan et al. (2018) indicated that *D.biguttusis*, *D.ellipicola*, *D.longicicola* and *D.mahothocarpus* should be treated as synonyms of *D.eres* using *cal*, *tef1* and *tub2* gene regions. In this study, we extended the work presented in Fan et al. (2018) and found seven additional strains belonging to *D.eres*. Additionally, the phylogenetic tree demonstrated that *D.camptothecicola* and *D.momicola* should also be treated as synonyms of *D.eres* (Fig. 2). *Diaporthecamptothecicola* from *Camptothecaacuminate* and *D.momicola* from *Prunuspersica* are described and illustrated based on the combined ITS, *cal*, *his3*, *tef1* and *tub2* regions (Dissanayake et al. 2017a, Yang et al. 2017c). Both of the two species are embedded in the *D.eres* complex. However, ITS analysis resulted in an unresolved phylogenetic tree without definitive bootstrap at the internodes, highly discordant to the trees resulting from the other four genes (Udayanga et al. 2014b). Therefore, the ITS region was not used in the combined analysis in the current study. To further investigate this complex, a second set of four (*cal*, *his3*, *tef1* and *tub2*), three (*cal*, *tef1* and *tub2*), two (*tef1* and *tub2*) and one (*tef1*) data matrices were performed following Santos et al. (2017) and Fan et al. (2018). The results showed that the three genes analyses (*cal*, *tef1* and *tub2*) appeared to be a better species recognition (Fig. 2). When it comes to this species complex, sequences supported by Udayanga et al. (2014b) are necessary to perform a more robust phylogenetic tree, clarifying the real species boundaries in this group in the future work.

#### Diaporthe fraxinicola

Taxon classificationFungiDiaporthalesDiaporthaceae

C.M. Tian & Q. Yang sp. nov.

MB824711

Figure 13

##### Diagnosis.

*Diaporthefraxinicola* can be distinguished from the closely related species *D.oraccinii* and *D.acerigena* (described above) based on ITS, *tef1* and *tub2* loci. *Diaporthefraxinicola* differs from *D.oraccinii* in larger alpha conidia and from *D.acerigena* in wider alpha conidia.

##### Holotype.

CHINA. Shaanxi Province: Zhashui city, Niubeiliang Reserve, on symptomatic twigs of *Fraxinuschinensis*, 7 July 2017, Q. Yang (holotype: BJFC-S1495; ex-type culture: CFCC 52582).

##### Etymology.

Named after the host genus on which it was collected, *Fraxinus*.

##### Description.

Conidiomata pycnidial, immersed in bark, scattered, slightly erumpent through the bark surface, nearly flat, discoid, with a single locule. Ectostromatic disc grey to dark brown, circular to ovoid, one ostiole per disc, 150–325 μm diam. Locule circular, undivided, 275–480 μm diam. Conidiophores 10.5–17.5 × 2.1–3.2 μm, hyaline, branched, cylindrical to clavate, straight, tapering towards the apex. Alpha conidia hyaline, aseptate, ellipsoidal to oval, 2–3-guttulate, rounded at both ends, 7–10 × 2.9–3.2 μm (av. = 8.5 × 3 μm, n = 30). Beta conidia hyaline, filiform, straight or hamate, eguttulate, aseptate, base subtruncate, tapering towards one apex, 19–29.5 × 1.4 µm (av. = 24.5 × 1.4 µm, n = 30).

**Figure 13.**
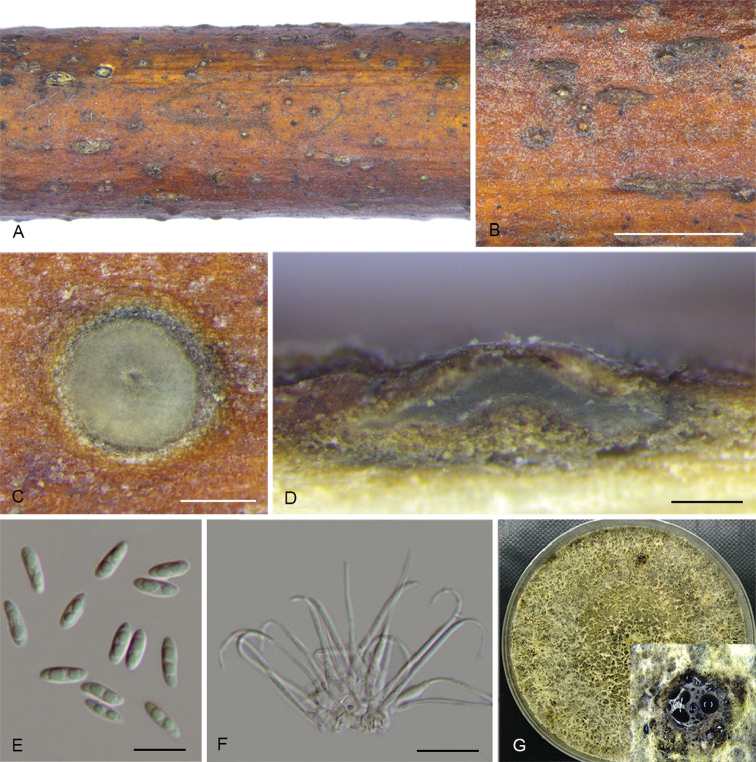
*Diaporthefraxinicola* (CFCC 52582) **A–B** Habit of conidiomata on branches **C** Transverse section of conidioma **D** Longitudinal section of conidioma **E** Alpha conidia **F** Beta conidia **G** Culture on PDA and conidiomata. Scale bars: 500 μm (**B**), 200 μm (**C**), 100 μm (**D**), 10 μm (**E–F**).

##### Culture characters.

Cultures incubated on PDA at 25 °C in darkness. Colony originally flat with white aerial mycelium, becoming yellowish, dense and felted aerial mycelium with age, with visible solitary or aggregated conidiomata at maturity.

##### Additional material examined.

CHINA. Shaanxi Province: Zhashui city, Niubeiliang Reserve, on symptomatic twigs of *Fraxinuschinensis*, 7 July 2017, Q. Yang, living culture CFCC 52583 (BJFC-S1496).

##### Notes.

This new species is introduced as molecular data, shows it to be a distinct clade with high support (ML/BI=100/1) and it appears most closely related to *D.oraccinii* and *D.acerigena*. *Diaporthefraxinicola* can be distinguished from *D.oraccinii* by 22 nucleotides in concatenated alignment, in which 6 were distinct in the ITS region, 8 in the *tef1* region and 8 in the *tub2* region; from *D.acerigena* by 27 nucleotides in concatenated alignment, in which 11 were distinct in the ITS region, 3 in the *tef1* region and 13 in the *tub2* region. Morphologically, *D.fraxinicola* differs from *D.oraccinii* in longer and larger alpha conidia (7–10 × 2.9–3.2 vs. 5.5–7.5 × 0.5–2 μm); differs from *D.acerigena* in larger alpha conidia (2.9–3.2 vs. 2.1–2.9 μm) (Gao et al. 2016).

#### Diaporthe kadsurae

Taxon classificationFungiDiaporthalesDiaporthaceae

C.M. Tian & Q. Yang sp. nov.

MB824713

Figure 14

##### Diagnosis.

*Diaporthekadsurae* differs from its closest phylogenetic species *D.fusicola* and *D.ovoicicola* in ITS, *cal* and *tef1* loci based on the alignments deposited in TreeBASE.

##### Holotype.

CHINA. Jiangxi Province: Shangrao city, Sanqing Mountain, on symptomatic branches of *Kadsuralongipedunculata*, 1 Apr. 2017, B. Cao, Y.M. Liang & C.M. Tian (holotype: BJFC-S1497; ex-type culture: CFCC 52586).

##### Etymology.

Named after the host genus on which it was collected, *Kadsura*.

##### Description.

Conidiomata pycnidial, immersed in bark, scattered, slightly erumpent through the bark surface, nearly flat, discoid, with a single locule. Ectostromatic disc obviously, brown to black, one ostiole per disc. Locule undivided, 475–525 μm diam. Conidiophores 7–11 × 1.8–2.9 μm, cylindrical, hyaline, unbranched, straight or slightly curved, tapering towards the apex. Alpha conidia hyaline, aseptate, oval or fusoid, biguttulate, 5.5–7.5 × 2.1–2.9 μm (av. = 6.5 × 2.5 μm, n = 30). Beta conidia not observed.

**Figure 14.**
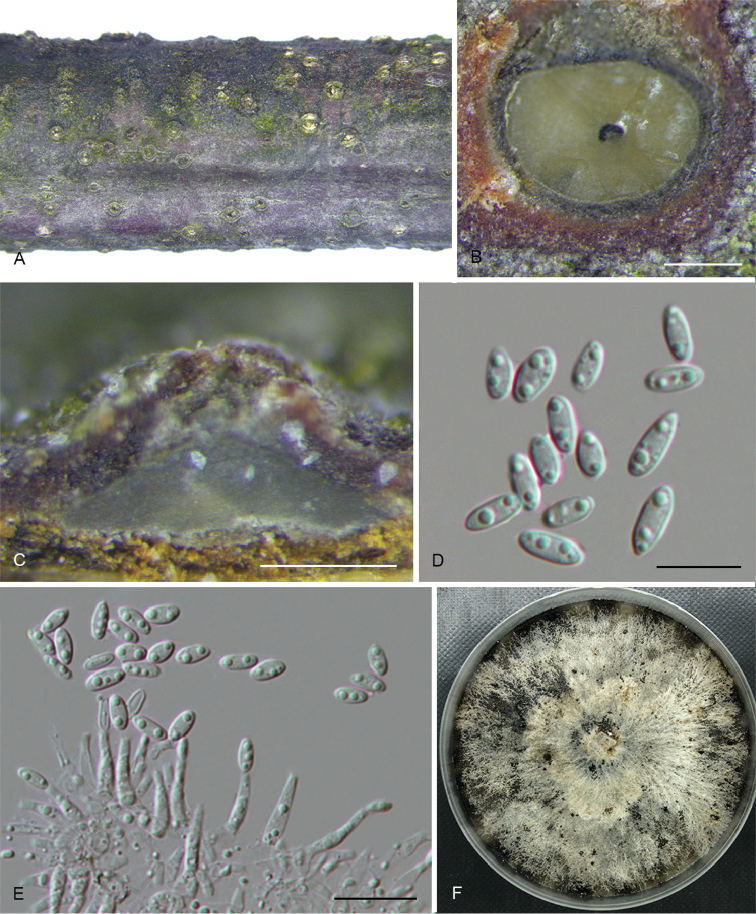
*Diaporthekadsurae* (CFCC 52586) **A** Habit of conidiomata on branches **B** Transverse section of conidioma **C** Longitudinal section of conidioma **D** Alpha conidia **E** Conidiophores **F** Culture on PDA. Scale bars: 200 μm (**B–C**), 10 μm (**D–E**).

##### Culture characters.

Cultures incubated on PDA at 25 °C in darkness. Colony originally flat with white aerial mycelium, becoming dense and felted aerial mycelium in the centre and grey to black mycelium at the marginal area with solitary conidiomata at maturity.

##### Additional specimens examined.

CHINA. Jiangxi Province: Shangrao city, Sanqing Mountain, on symptomatic branches of *Kadsuralongipedunculata*, 1 Apr. 2017, B. Cao, Y.M. Liang & C.M. Tian, living culture CFCC 52587 (BJFC-S1498); Yunbifeng National Forest Park, on symptomatic twigs of *Acer* sp., 31 Mar. 2017, B. Cao, Y.M. Liang & C.M. Tian, living culture CFCC 52588 (BJFC-S1499); ibid. living culture CFCC 52589 (BJFC-S1500).

##### Notes.

This new species is introduced as molecular data show it to be a distinct clade with high support (ML/BI=100/1) and it appears most closely related to *D.fusicola* and *D.ovoicicola*. *Diaporthekadsurae* can be distinguished from *D.fusicola* by 11 nucleotides in concatenated alignment, in which 4 were distinct in the ITS region and 7 in the *cal* region; from *D.ovoicicola* by 25 nucleotides in concatenated alignment, in which 12 were distinct in the ITS region, 6 in the *cal* region and 7 in the *tef1* region. Morphologically, *D.kadsurae* differs from *D.fusicola* and *D.ovoicicola* in shorter conidiophores (7–11 μm in *D.kadsurae* vs. 11–24.1 μm in *D.fusicola*; 7–11 μm in *D.kadsurae* vs. 14.2–23.6 μm in *D.ovoicicola*) (Gao et al. 2014).

#### Diaporthe padina

Taxon classificationFungiDiaporthalesDiaporthaceae

C.M. Tian & Q. Yang sp. nov.

MB824714

Figure 15

##### Diagnosis.

*Diaporthepadina* can be distinguished from the phylogenetically closely related species *D.betulae* in smaller conidiomata and alpha conidia.

##### Holotype.

CHINA. Heilongjiang Province: Liangshui Nature Reserve, on symptomatic twigs of *Padusracemosa*, 31 July 2016, Q. Yang (holotype: BJFC-S1501; ex-type culture: CFCC 52590).

##### Etymology.

Named after the host genus on which it was collected, *Padus*.

##### Description.

Conidiomata pycnidial, immersed in bark, scattered, slightly erumpent through the bark surface, discoid, with a single locule. Ectostromatic disc light brown, one ostiole per disc, 330–520 μm diam. Locule circular, undivided, 250–550 μm diam. Conidiophores 5.5–12.5 × 1–1.5 μm, hyaline, unbranched, cylindrical, straight or slightly curved. Alpha conidia hyaline, aseptate, ellipsoidal to fusiform, eguttulate, 7–8 × 1.5–2 μm (av. = 7.5 × 1.8 μm, n = 30). Beta conidia hyaline, filiform, straight or hamate, eguttulate, aseptate, base truncate, 21–24 × 1 µm (av. = 22 × 1 µm, n = 30).

**Figure 15.**
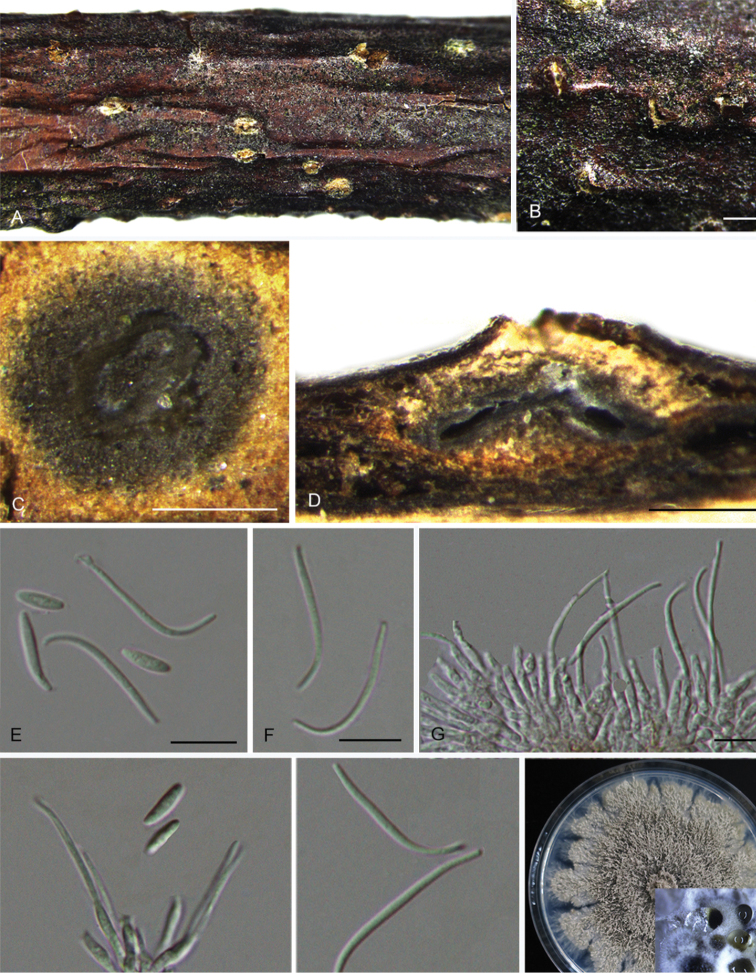
*Diaporthepadina* (CFCC 52590) **A–B** Habit of conidiomata on branches **C** Transverse section of conidioma **D** Longitudinal section of conidioma **E** Alpha and beta conidia **F, I** Beta conidia **G–H** Conidiophores **J** Culture on PDA and conidiomata. Scale bars: 500 μm (**B**), 200 μm (**C–D**), 10 μm (**E–I**).

##### Culture characters.

Cultures incubated on PDA at 25 °C in darkness. Colony originally flat with white aerial mycelium, becoming grey to brown in the centre, with pale grey, felted, valviform mycelium at the marginal area and aggregated conidiomata at maturity.

##### Additional material examined.

CHINA. Heilongjiang Province: Liangshui Nature Reserve, on symptomatic twigs of *Padusracemosa*, 31 July 2016, Q. Yang, living culture CFCC 52591 (BJFC-S1502).

##### Notes.

Four strains representing *D.padina* cluster in a well-supported clade and appear closely related to *D.betulae*. This species is phylogenetically closely related to, but clearly differentiated from, *D.betulae* by 40 different unique fixed alleles in ITS, *cal*, *his3*, *tef1* and *tub2* loci (4, 7, 10, 13 and 6 respectively) based on the alignments deposited in TreeBASE. Morphologically, *D.padina* differs from *D.betulae* in smaller conidiomata and alpha conidia (250–550 vs. 600–1250 μm in conidiomata; 7–8 × 1.5–2 vs. 8.5–11 × 3–4 μm in alpha conidia) (Du et al. 2016).

#### Diaporthe ukurunduensis

Taxon classificationFungiDiaporthalesDiaporthaceae

C.M. Tian & Q. Yang sp. nov.

MB824715

Figure 16

##### Diagnosis.

*Diaportheukurunduensis* can be distinguished from the phylogenetically closely related species *D.citrichinensis* in longer conidiophores and shorter alpha conidia.

##### Holotype.

CHINA. Shaanxi Province: Qinling Mountain, on symptomatic twigs of *Acerukurunduense*, 27 June 2017, Q. Yang (holotype: BJFC-S1503; ex-type culture: CFCC 52592).

##### Etymology.

Named after the host species on which it was collected, *Acerukurunduense*.

##### Description.

Conidiomata pycnidial, immersed in bark, serried, slightly erumpent through the bark surface, nearly flat, discoid, with a single locule. Ectostromatic disc dark brown to black, one ostiole per disc. Locule circular, undivided, 165–215 μm diam. Conidiophores 11.5–18 × 1.5 μm, hyaline, branched, cylindrical, straight or curved. Alpha conidia hyaline, aseptate, ellipsoidal to oval, biguttulate, 5–6 × 2.1–2.9 μm (av. = 5.5 × 2.5 μm, n = 30). Beta conidia not observed.

**Figure 16.**
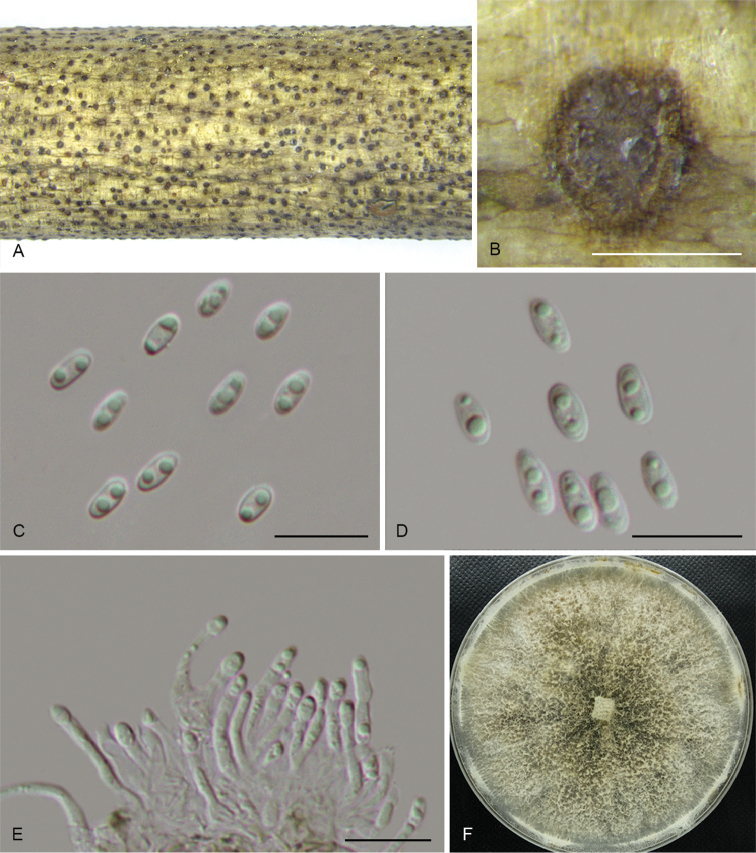
*Diaportheukurunduensis* (CFCC 52592) **A** Habit of conidiomata on branches **B** Transverse section of conidioma **C–D** Alpha conidia **E** Conidiophores **F** Culture on PDA. Scale bars: 200 μm (**B**), 10 μm (**C–E**).

##### Culture characters.

Cultures incubated on PDA at 25 °C in darkness. Colony originally flat with white aerial mycelium, becoming brown to pale black in the centre, dense, felted, conidiomata not observed.

##### Additional specimens examined.

CHINA. Shaanxi Province: Qinling Mountain, on symptomatic twigs of *Acerukurunduense*, 27 June 2017, Q. Yang, living culture CFCC 52593 (BJFC-S1503).

##### Notes.

*Diaportheukurunduensis* comprises strains CFCC 52592 and CFCC 52593 closely related to *D.citrichinensis* in the combined phylogenetic tree (Fig. 1). *Diaportheukurunduensis* can be distinguished from *D.citrichinensis* based on ITS and *tef1* loci (10/470 in ITS and 4/336 in *tef1*).

#### Diaporthe unshiuensis

Taxon classificationFungiDiaporthalesDiaporthaceae

F. Huang, K.D. Hyde & H.Y. Li, 2015

Figure 17

##### Description.

On PNA: Conidiomata pycnidial, globose or rostrated, black, erumpent in tissue, erumpent at maturity, 260–500 μm diam, often with translucent conidial drops exuding from the ostioles. Conidiophores 18–28.5 × 1.4–2.1 μm, cylindrical, hyaline, branched, septate, straight or curved, tapering towards the apex. Alpha conidia abundant in culture, hyaline, aseptate, ellipsoidal to fusiform, biguttulate, sometimes with one end obtuse and the other acute, 6.5–8.5 × 2.1–2.5 μm (av. = 7.8 × 2.3 μm, n = 30). Beta conidia not observed.

**Figure 17.**
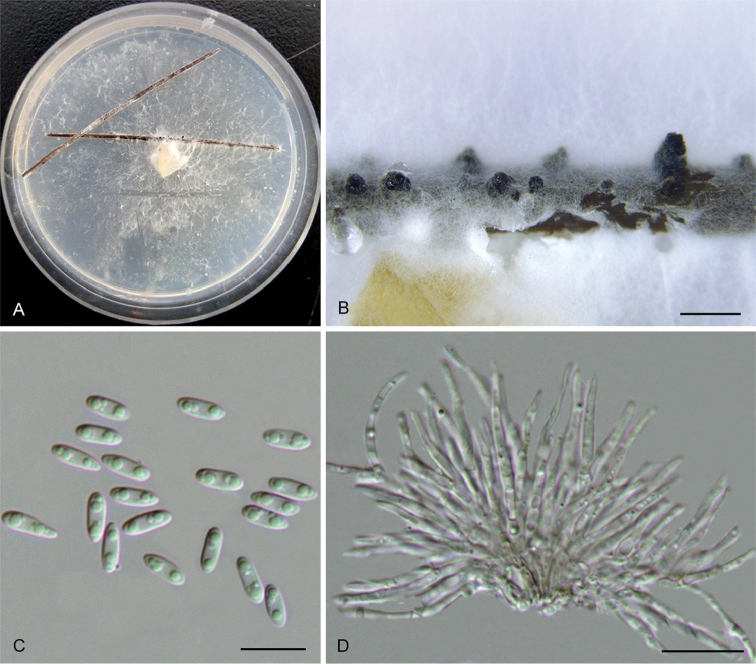
*Diaportheunshiuensis* (CFCC 52594) **A** Culture on PNA **B** Conidiomata **C** Alpha conidia **D** Conidiophores. Scale bars: 500 μm (**B**), 10 μm (**C–D**).

##### Culture characters.

Cultures incubated on PNA at 25 °C in darkness. Colony entirely white at surface, reverse with pale brown pigmentation, white, fluffy aerial mycelium.

##### Specimens examined.

CHINA. Jiangsu Province: Nanjing city, on non-symptomatic twigs of *Caryaillinoensis*, 10 Nov. 2015, Q. Yang, living culture CFCC 52594 and CFCC 52595 (BJFC-S1476).

##### Notes.

*Diaportheunshiuensis* was originally described from twigs of non-symptomatic *Fortunellamargarita* in Zhejiang Province, China (Huang et al. 2015). In the present study, two isolates from twigs of asymptomatic *Caryaillinoensis* were congruent with *D.unshiuensis* based on morphology and DNA sequences data (Fig. 1). We therefore describe *D.unshiuensis* as a known species for this clade.

## Discussion

The current study described 15 *Diaporthe* species from 42 strains based on a large set of freshly collected specimens. It includes 12 new species and 3 known species, which were sampled from 16 host genera distributed over six Provinces of China (Table 1). In this study, 194 reference sequences (including outgroup) were selected based on BLAST searches of NCBIs GenBank nucleotide database and included in the phylogenetic analyses (Table 1). Phylogenetic analyses based on five combined loci (ITS, *cal*, *his3*, *tef1* and *tub2*), as well as morphological characters, revealed the diversity of *Diaporthe* species in China, mainly focusing on diebacks from major ecological or economic forest trees.

Several studies have been conducted associated with various hosts in China. For instance, the research conducted by Huang et al. (2015) revealed seven apparently undescribed endophytic *Diaporthe* species on *Citrus*. Gao et al. (2016) demonstrated that *Diaporthe* isolates, associated with *Camellia* spp., could be assigned to seven species and two species complexes. Recently, *Diaporth*e has been revealed as paraphyletic by Gao et al. (2017), showing that *Ophiodiaporthe*, *Pustulomyces*, *Phaeocytostroma* and *Stenocarpella* embed in *Diaporthes. lat.* and eight new species of *Diaporthe* were introduced from leaves of several hosts. However, the identification of *Diaporthe* species associated with dieback of forest trees has rarely been studied, thus a large-scale investigation of *Diaporthe* spp. was conducted from 2015 to 2017. This study provides the first molecular phylogenetic frame of *Diaporthe* diversity associated with dieback in China, combined with morphological descriptions.

*Diaportheeres*, the type species of the genus, was initially described by Nitschke (1870), from *Ulmus* sp. collected in Germany. The major problem with this generic type was the lack of an ex-type culture or ex-epitype culture, although a broad species concept has historically been associated with *D.eres* (Udayanga et al. 2014b). Udayanga et al. (2014b) designed strain AR5193 as the epitype of *D.eres* and provided the phylogram of this complex using seven loci (ITS, *act*, Apn2, *cal*, *his3*, FG1093, *tef1* and *tub2*), amongst which the *tef1*, Apn2 and *his3* genes were recognised as the best markers for defining species in the *D.eres* complex. Moreover, they showed that poorly supported non-monophyletic grouping was observed when ITS sequences were included in the combined analysis. In this study, although we conducted phylogenetic analysis as performed in previous studies on *Diaporthe* species (Santos et al. 2017), much confusion has, however, occurred in species separation of *the D.eres* complex (Fig. 1). Especially, the ITS region could lead to a confused taxonomic situation within this species complex. We found the three-gene analysis, excluding the ITS and *his3* regions, resulted in a more robust tree congruent with Udayanga et al. (2014b) and resolved the species boundaries within the *D.eres* species complex. The isolates, clustering with *D.eres* in this study, occur on multiple hosts from many different geographic locations. This study revealed three new species belonging to the *D.eres* complex, i.e. *D.betulina*, *D.chensiensis* and *D.padina*. It also shows *D.biguttusis*, *D.camptothecicola*, *D.ellipicola*, *D.longicicola*, *D.mahothocarpus* and *D.momicola* were clustered in *D.eres* and should be treated as synonyms of *D.eres*, which is in conformity with Fan et al. (2018).

The initial species concept of *Diaporthe*, based on the assumption of host-specificity, resulted in the introduction of more than 1000 taxa (http://www.indexfungorum.org/). Thus, during the past decade, a polyphasic approach, employing multi-locus DNA data together with morphology and ecology, has been employed for species boundaries in the genus (Crous et al. 2012, Udayanga et al. 2014a, b, Huang et al. 2015, Gao et al. 2016, 2017, Guarnaccia and Crous 2017, 2018, Hyde et al. 2017, 2018, Yang et al. 2017a, b, 2018, Guarnaccia et al. 2018, Jayawardena et al. 2018, Perera et al. 2018a, b, Tibpromma et al. 2018, Wanasinghe et al. 2018).

Further studies are required in order to conduct an extensive collection of *Diaporthe* isolates, to resolve taxonomic questions and to redefine species boundaries. Multiple strains from different locations should also be subjected to multi-gene phylogenetic analysis to determine intraspecific variation. The descriptions and molecular data of *Diaporthe* species provided in this study represent a resource for plant pathologists, plant quarantine officials and taxonomists for identification of *Diaporthe*.

## Supplementary Material

XML Treatment for Diaporthe acerigena

XML Treatment for Diaporthe alangii

XML Treatment for Diaporthe betulina

XML Treatment for Diaporthe biguttulata

XML Treatment for Diaporthe caryae

XML Treatment for Diaporthe cercidis

XML Treatment for Diaporthe chensiensis

XML Treatment for Diaporthe cinnamomi

XML Treatment for Diaporthe conica

XML Treatment for Diaporthe eres

XML Treatment for Diaporthe fraxinicola

XML Treatment for Diaporthe kadsurae

XML Treatment for Diaporthe padina

XML Treatment for Diaporthe ukurunduensis

XML Treatment for Diaporthe unshiuensis
